# In Silico and In Vitro development of novel small interfering RNAs (siRNAs) to inhibit SARS-CoV-2

**DOI:** 10.1016/j.csbj.2025.03.034

**Published:** 2025-03-23

**Authors:** Noha Samir Taibe, Sara H. Mahmoud, Maimona A. Kord, Mohamed Ahmed Badawy, Mahmoud Shehata, Mahmoud Elhefnawi

**Affiliations:** a Biotechnology Department, Faculty of Science, Cairo University, Giza, Egypt; b Center of Scientific Excellence for Influenza Viruses (CSEIV), National Research Centre, Cairo 12622, Egypt; c Botany Department, Faculty of Science, Cairo University, Giza, Egypt; d Chemistry Department, Faculty of Science, Cairo University, Giza, Egypt; e Biomedical Informatics and Cheminformatics Group, Informatics and Systems Department, National Research Centre, Cairo, Egypt

**Keywords:** SARS-CoV-2, Therapy, Silencing, SiRNA, Non-structure proteins (NSP), Open reading frame(ORF)

## Abstract

SARS-CoV-2 is causing severe to moderate respiratory tract infections, posing global health, social life, and economic threats. Our design strategy for siRNAs differs from existing studies through a step-by-step filtration process utilizing integrative bioinformatics protocols and web tools. Stage one: Multiple Sequence Alignment was employed to identify the most conserved areas. Stage two involves using various online tools, among the most reputable tools for building siRNA. The first filtration step of siRNA uses the Huesken dataset, estimating a 90 % experimental inhibition. The second filtration stage involves choosing the most suitable and targeted siRNA by utilizing thermodynamics and Target Accessibility of siRNAs. The final filtration step is off-target filtration using BLAST with specific parameters. Four of the 258 siRNAs were chosen for their potency and specificity, targeting conserved regions (NSP8, NSP12, and NSP14) with minimal human transcripts off-targets. We conducted in-vitro experiments, including cytotoxicity, TCID50, and RT-PCR assays. When tested on the SARS-CoV-2 strain hCoV-19/Egypt/NRC-03/2020 at 100 nM, none showed cellular toxicity. The TCID50 assay confirmed viral replication reduction at 12 h.p.i; the efficacy of the four siRNAs and their P value were highly significant. siRNA2 maintaining efficacy at 24, 36, and 48 h.p.i, while siRNA4 had a significant P value (≤0.0001) at 48 h.p.i. At 24 h.p.i, siRNA2 and siRNA4 showed statistical significance in viral knockdown of the virus's S gene and ORF1b gene by 95 %, 89 %, and 96 %, 97 %, respectively. Our computational method and experimental assessment of specific siRNAs have led us to conclude that siRNA2 and siRNA4 could be promising new therapies for SARS-CoV-2 that need further development.

## Graphical Abstract

**Figure:**
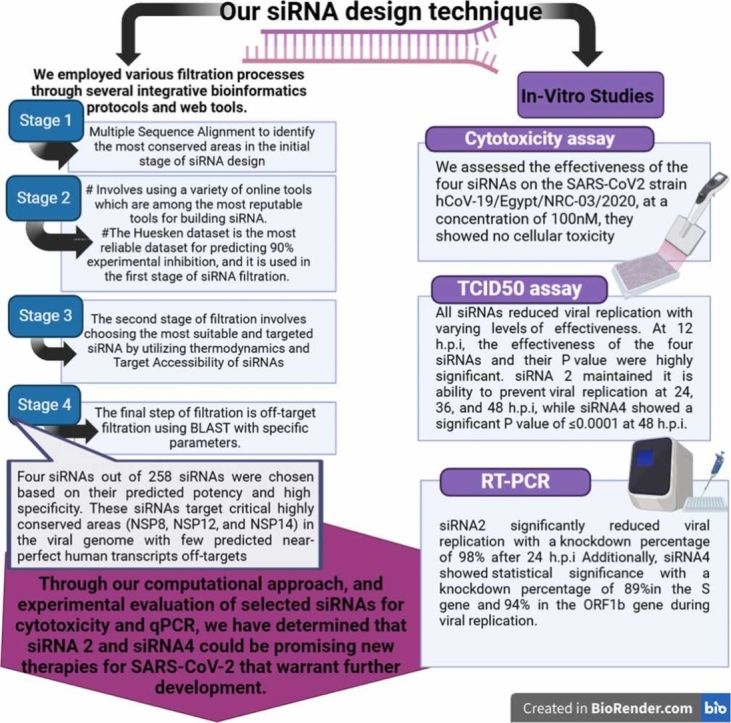

## 1 Introduction

In the last 30 years, three new Beta-coronaviruses, namely Severe Acute Respiratory Syndrome (SARS)-CoV, Middle East Respiratory Syndrome (MERS)-CoV, and SARS-CoV-2, have emerged and caused significant outbreaks in humans with high case-fatality rates[1], [2]. SARS-CoV-2, the cause of COVID-19 (Corona Virus Infectious Disease – 2019), is the most recent addition to human pathogenic coronaviruses (HCoVs)[3]. Among positive-stranded RNA (+RNA) viruses, coronaviruses (CoVs) are a group of zoonotic viruses that are enveloped and non-segmented, with the largest genome (26–32 kb).[4]. During discontinuous negative-strand RNA synthesis, the leader and 'body' segments of the sg(subgenomic) RNAs are joined, yielding a subgenome-length template for each sg mRNA [5], [6].

**Fig. 1:**
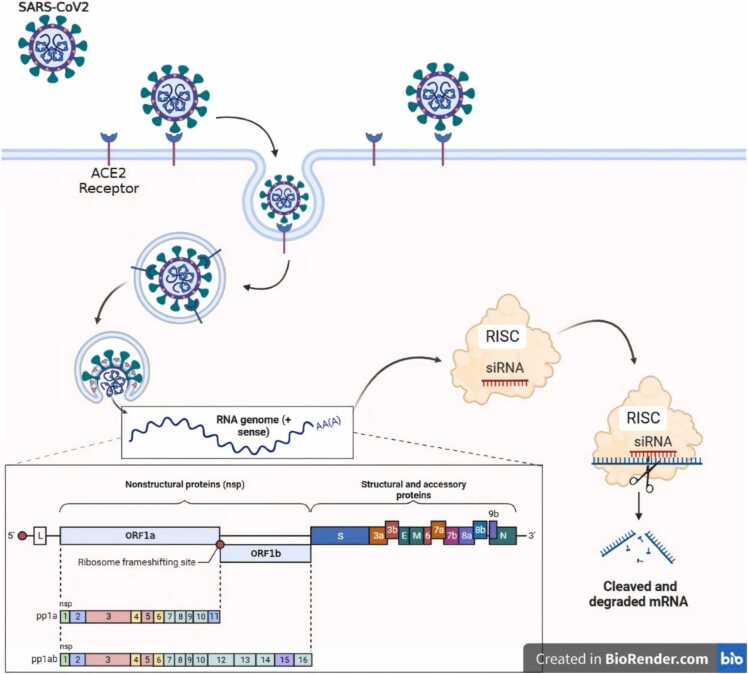
**Schematic representation of the siRNA gene silencing mechanism.** SARS-CoV-2 binds to the surface receptor angiotensin-converting enzyme 2 (ACE-2) on the host cell and releases its RNA genome in the cytoplasm via endocytosis or direct membrane fusion. After siRNA transfection using Lipofectamine3000, the active RISC guides the guide strand of siRNA to the target viral genomic RNA. Once the guide strand of siRNA binds to the target region of viral mRNA, this leads to the cleavage of the viral mRNA. Created with BioRender.

Coronaviruses are positive single-stranded RNA viruses that use ORF1a and ORF1b replicases, as shown in Fig. 1, making RNA interference (RNAi) a potentially effective therapeutic approach for controlling virus replication by targeting viral mRNA, limiting the synthesis of the proteins necessary for viral replication, obstructing viral cellular entry and trafficking at particular stages in human cells[7], [8]. mRNA-based vaccination techniques helped pave the path for a new age of RNA therapies during the SARS-CoV-2 pandemic. Small interfering RNA-based methods relying on RNAi may supplement clinical COVID-19 care and enable the development of potent antivirals to lessen COVID-19 pathogenesis [9], [10]. Medeiros IG et al.[11] indicated a database of each possible 18–21 nucleotide siRNA target region from SARS-CoV-2. Synthetic siRNAs function, which have a length of 18–25 base pairs, is to block a specific post-transcriptional gene expression[11]. The RNAi approach can be based either on the introduction of siRNA into cells of lengthy dsRNAs (double-stranded RNAs) that are subsequently cut into short dsRNAs of around twenty-one base pairs by RNase III (Dicer) in the cytoplasm to form siRNA as shown in Fig. 1[12] or on the direct delivery of siRNA through liposomes, viral vectors, aptamers, and cell-penetrating peptides[13].

Computational methods and machine learning[14], [15] are used to build well-targeted and precise siRNAs after first retrieving the genomes of the SARS-CoV-2 virus and its variants. Multiple sequence alignment is used to identify genomic regions conserved across different variants[7]. These conserved areas between variants are typically considered potential siRNA target locations. The design of siRNAs(as implemented here and elsewhere)is carried out using web servers, incorporating the results of different algorithms based on Ui-Tei[16], Amarzguioui[17], and Reynolds[18] criteria.

For SARS-CoV-2, siRNA prediction, synthesis, and design have been pioneered by numerous in silico studies. Pandey and Verma, 2021[19] conducted a study using in silico methods to target the virus leader sequence. Shawan, M.M.A.K., et al.,created siRNA sequences directed at the SARS-CoV-2 RNA-dependent RNA polymerase (RdRp) gene through docking and molecular dynamics modeling to evaluate how well each siRNA sequence bound to the RdRp gene segment[20].

In this study, we developed four siRNAs with high potency and specificity targeting non-structural proteins (NSP8, NSP12, and NSP14) of human pathogenic coronaviruses, including SARS-CoV, MERS-CoV, and SARS-CoV-2. We carefully considered various factors, such as RNA variations, thermodynamics, accessibility, and off-target effects, to enhance RNAi efficiency and minimize potential side effects. However, due to the urgent need to combat the pandemic caused by SARS-CoV-2, we specifically tested the siRNAs targeting this virus.

Up to 48 h.p.i (hours post-infection), two siRNAs were shown to dramatically knock down viral genes and reduce viral titers, making them promising candidates for additional in vivo testing as potential therapeutics for SARS-CoV-2.

## 2 Materials and methods

### 2.1 In-silico design of siRNAs

**Fig. 2:**
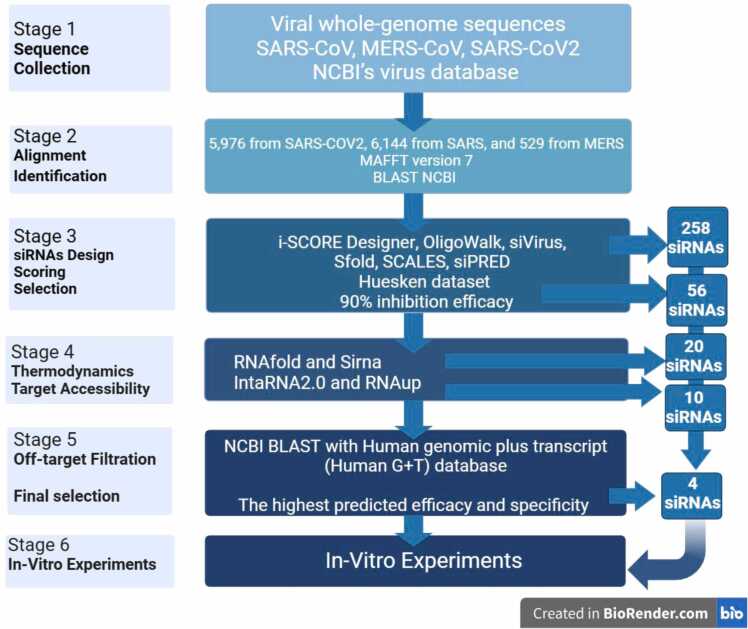
**Flowchart of in-silico siRNA design steps and methodology.** Stages 1: All SARS-Cov, MERS-Cov, and SARS-Cov-2 viral genome sequences up to 7.5.2022 were downloaded from NCBI’s Virus database. Stage 2: A total of 5976 sequences from SARS-CoV-2, 6144 from SARS, and 529 from MERS were aligned using MAFFT version 7. Conserved regions resulting from the alignment were identified through BLAST NCBI. Stage 3: 258 siRNAs were designed using various online software such as i-SCORE Designer, OligoWalk, siVirus, Sfold, SCALES, and siPRED. Based on the Huesken dataset and 90 % experimental threshold for inhibition scores, 56 siRNAs were selected. Stage 4: Twenty siRNAs were identified after analyzing thermodynamic features with RNAfold and Sirna. Additionally, ten siRNAs were chosen based on target accessibility using RNAup and IntaRNA. Stage 5: In the final filtration step, any siRNA with a perfect match to the Human genomic plus transcript (Human G+T) database was rejected using NCBI BLAST. Four siRNAs were ultimately selected for in vitro testing. Created with BioRender.

Our integrated approach to creating siRNAs was based on the methods previously published in [21] [22] and[23]. Our workflow consists of six stages, as illustrated in Fig. 2. Stage 1 involves gathering viral sequences from the NCBI virus database; Stage 2 includes multiple sequence alignment of sequences and identification of resultant conserved sequences; Stage 3 consists of designing siRNAs using various online tools, followed by scoring and siRNA selection; Stage 4 calculates the thermodynamic properties of siRNAs and the target accessibility using multiple online tools; and Stage 5 involves filtering off-target siRNAs and selecting the final set of siRNAs that will proceed to the sixth stage of the study, which is in vitro experiments. The characteristics and thermodynamic properties of our designed siRNAs used in this study are presented in Table 1.

**Table 1:** Features of siRNAs validated.

Name	siRNA sequence(sense)*^1^	Target*^2^	Position *^3^	Thermodynamic properties * 4	Target accessibility * 5
siRNA 1	5`G.G.A.C.A.A.G.A.G.G.G.C.A.A.A.A.G.U.U.au3`	NSP8	12239:12258	−31.1	−37.46
siRNA 2	5`G.A.C.A.A.G.A.G.G.G.C.A.A.A.A.G.U.U.A.au3`	NSP8	12282:12305	−28.7	−35.56
siRNA 3	5`G.G.C.U.U.U.G.A.G.U.U.G.A.C.A.U.C.U.A.au3`	NSP14	18565:18586	−29.7	−33.38
siRNA 4	5`G.U.C.U.A.A.G.G.G.U.U.U.C.U.U.U.A.A.G.au3`	NSP12	14674:14699	−26.2	−27.74

* 1 siRNA sequence targeting conserved regions.

* 2 conserved regions.

* 3 Position on the reference sequence.

* 4 Whole ΔG.

* 5 total free energy of binding.

### 2.2 Sequence Collection

Our attention's focus was on the three most common coronavirus species classified as human pathogenic coronaviruses, SARS-CoV, Middle East Respiratory Syndrome (MERS)-CoV, and SARS-CoV-2, the source of COVID-19. On 5/7/2020, viral whole-genome sequences were obtained from NCBI’s virus database https://www.ncbi.nlm.nih.gov/labs/virus/vssi/#/.

### 2.3 Multiple sequence alignment and identification of conserved regions

12,649 sequences (5976 from SARS-CoV-2, 6144 from SARS-CoV, and 529 from MERS-CoV) were used in a multiple sequence alignment process conducted using MAFFT version 7 [24]. The alignment output file was analyzed with Jal view [25] to identify the most conserved region at a percentage identity threshold of 86.4 %. As indicated in Table 2, The SARS-CoV-2 reference genome sequence (NC 045512) was used to determine the resulting conserved areas through BLAST NCBI https://blast.ncbi.nlm.nih.gov/. The resulting conserved regions were aligned against different SARS-CoV-2 variants as Alpha Beta, Delta, Epsilon, Eta, Gamma, Lota, Kappa, Lambda, Zeta, Omicron BA.1, Omicron BA.2, Omicron BA.2.12.1, Omicron BA.2.75, Omicron BA.4, Omicron BA.5, Omicron BQ.1.1, Omicron EG.5.1, Omicron XBB.1.5 using Clustal Omega Clustal Omega < Multiple Sequence Alignment < EMBL-EBI. The output file for alignment was viewed using Jalview[25].

**Table 2:** The final conserved regions,the name of the equivalent protein, and the nucleotide position.

NO.	Conserved regions	Blast region	Nucleotide position
1	ATGGCTGATCAAGCTATGAC	NSP8 in ORF1ab	12239:12258
2	CTGAGGACAAGAGGGCAAAAGTTA	NSP8 in ORF1ab	12282:12305
3	GTGTCTAAGGGTTTCTTTAAGGAAGG	RNA-dependent RNA polymerase (NSP12)	14674:14699
4	TTCTTTGCTCAGGATGGTAATGCTGCTAT	RNA-dependent RNA polymerase (NSP12)	14718:14742
5	GTGTCTTTAGCTATAGATGCTTACCC	RNA-dependent RNA polymerase (NSP12)	15982:16007
6	TTTACTGGTTATCGTGTAAC	ORF1ab Helicase (NSP13)	16733:16752
7	GTCTTTATTTCACCTTATAATTCACAGAATGCTGTAGC	ORF1ab Helicase (NSP13)	17710:17747
8	CAATTTAAACACCTCATACC	ORF1ab 3′:5′ exonuclease (NSP14)	18425:18444
9	CATGGCTTTGAGTTGACATCTA	ORF1ab 3′:5′ exonuclease (NSP14)	18565:18586
10	AAATGGCCATGGTACATTTGGCT	gene S prod: surface glycoprotein	25146:25168

### 2.4 siRNA Design, scoring, and selection steps

All possible siRNAs for the conserved regions were designed using various online software programs such as i-SCORE Designer[26], OligoWalk [27], siVirus [28], Sfold [29], SCALES [30], and siPRED[31]. The Huesken dataset[32] was used as the first filter to obtain siRNAs, resulting from i-SCORE Designer using a linear regression model by IBM SPSS Statistics, with a predicted experimental inhibition of 90 % to filter the siRNAs. Threshold scores were assigned to the software programs to establish an accepted threshold as indicated in Additional File 1, and the best siRNAs were selected based on surpassing all prior thresholds.

### 2.5 Thermodynamics and Target Accessibility

As the stability of siRNA duplex is crucial, RNAfold[33], and Sirna[29] were utilized to calculate RNA duplex thermodynamics for the designed siRNA and the difference in the 5′ end terminal free energy (ddG) of the sense and antisense strands. siRNAs with dG values ranging from −35 to −27 kcal/mol were chosen [34]. The secondary structure of the siRNA with target areas in the SARS-CoV-2 mRNA was determined using Sfold[29] and RNAfold[33]. IntaRNA2.0[35] and RNAup[33] were employed to ensure the designed siRNAs could reach their targets.

### 2.6 Off-target filtration and final selection for designed siRNAs

Using parameters from the classical Birmingham protocol[22], any near-complete or seed region matches with other crucial genes that could lead to downregulation of these genes were avoided by comparing the seed region and complete sequence of siRNAs with Human genomic plus transcript (Human G+T) database in NCBI BLAST [36]. The siRNAs with the highest anticipated efficacy and specificity were chosen for experimental testing following each filtration stage., as indicated in Additional File 1.

### 2.7 In vitro methods

#### 2.7.1 siRNAs preparation

The synthesis of the selected siRNAs, including a positive control siRNA (targeting GAPDH)5-GUAUGACAACAGCCUCAAGTT-3(forward) and 5_CUUGAGGCUGUUGUCAUACTT-3 (reverse), and a negative control siRNA (scrambled) 5-UUCUCCGAACGUGUCACGUTT-3(forward) and 5-ACGUGACACGUUCGGAGAATT-3(reverse)[37] was conducted by Dharmacon, Horizon Discovery, UK. Our procedure for preparing and aliquoting siRNAs followed Horizon Discovery's (UK) siRNA resuspension protocol and then stored at −20°C.

#### 2.7.2 Virus propagation

Vero E6 cells were cultured at 37°C with 5 % CO_2_ in Dulbecco's modified Eagle's medium (DMEM) supplemented with 10 % fetal bovine serum (FBS) and 1 % penicillin/streptomycin (pen/strep) antibiotic combination. SARS-CoV-2 isolate(hCoV-19/Egypt/NRC-03/2020 GISAID accession number: EPI ISL 430819) propagated in Vero E6 cells[34]. All SARS-CoV-2 infection experiments were conducted under biosafety level-3 conditions (BSL-3).

#### 2.7.3 Cytotoxicity (CC50) determination

The siRNAs were dissolved in 1x siRNA buffer (catalog ID: B-002000-UB-100, Dharmacon, Horizon Discovery, UK) at a concentration of 10 µM to determine the CC50. The siRNA stock solution was further diluted to working solutions (10 dilutions) using DMEM (10 µM - 0.0195 µM) as previously described in [38]. The crystal violet assay was used to assess cytotoxicity in VERO-E6 cells.[38]. To measure CC50 (half-maximal cytotoxic concentration) of siRNAs, the siRNAs were serially diluted (Bi-fold dilutions) in DMEM medium.

Briefly, the cells were seeded in 96-well plates (100 μl/well at a density of 3 ×10^5^ cells/ml) and incubated at 37°C in 5 % CO2 for 24 hours. After 24 hours, the cells were treated in triplicate with different doses of siRNAs, and untreated cells were used as a control. The supernatant was discarded 72 hours after treatment, and cell monolayers were fixed with 10 % formaldehyde for 1 hour at room temperature (RT). The fixed monolayers were adequately dried before being stained with 50 µl of 0.1 % crystal violet on a bench rocker at room temperature for 20 minutes. After washing and drying the monolayers overnight, the crystal violet dye in each well was dissolved in 200 µl methanol for 20 minutes on a bench rocker at room temperature. The absorbance of the crystal violet solutions was measured at λmax 570 nm as a reference wavelength using a multiwell plate reader. The CC_50_ value was calculated using nonlinear regression analysis using GraphPad Prism software (version 5.01) by plotting log concentrations of the compound versus normalized response (variable slope). The percentage of cytotoxicity compared to the untreated cells was determined using the following equation:

Cytotoxicity %= (absorbance of cells without treatment-absorbance of cells with treatment)/ (absorbance of cells without treatment) X 100

The CC50 (50 % cytotoxicity) concentration was calculated using a plot of percent cytotoxicity vs sample concentration[39].

#### 2.7.4 Virus propagation and titration

SARS-CoV-2 strain hCoV-19/Egypt/NRC-03/2020 (GISAID accession number: EPI_ISL_430819) was propagated in Vero-E6 cells to generate virus stock in the presence of L-1-tosylamido-2-phenylethyl chloromethyl ketone (TPCK)-treated trypsin. The virus stock was propagated in Vero-E6 cells cultured at a multiplicity of infection (MOI) of 0.001, and then the cells were microscopically investigated daily. The Vero E6 cells infected with SARS-CoV-2 isolate were used as a control, and Vero E6 cells were used as an untreated control. The virus-infected culture supernatant was clarified by centrifugation at 4000 rpm for 15 min at 4°C twice. The harvested virus was titrated by TCID_50_ assay.

#### 2.7.5 Titration of virus (TCID50 assay)

The hCoV-19/Egypt/NRC-03/2020 SARS-CoV-2 isolate virus was serially diluted (half log dilution from neat to log7.5) using a 96-well dilution plate. A total of 230 µl of DMEM with 2 % BSA (Bovine Serum Albumin) (infection media) was added to the wells (from A to G in column 1 and from A to H in column 5 b). Then, 300 µl of the virus was added to column 1 row H. 105 μl of the virus was transferred from row H column 1 to row G column 1. This process was repeated until log 7.5 was achieved, with the series dilution being repeated each time with a change of filter pipette tips. One day before transfection, 96-well plates were seeded with Vero cells. The cell culture plates were transfected with 100 nM of six siRNAs following the Basic siRNA Resuspension Protocol of Dharmacon, Horizon Discovery, UK according to the Lipofectamine 3000 protocol. After 24 hours, the 96-well Vero-E6 confluent cell plates were prepared by washing the cells two times with an infection medium. The plates were infected with an MOI of 0.01 of the virus, and 35 µl of each viral dilution was applied to Vero-E6 cells in 96 well microtiter dilution plates in quadruplicate with cell controls kept in the same plate. The plates were then incubated for 1 hr at 37ᴼC in a 5 % CO_2_ incubator, the virus dilutions were removed, and the plates were washed with 1X PBS. Subsequently, 150 μl of infection medium was added to the Vero cell plate which was then incubated at 37ᴼC in a 5 % CO_2_ incubator for 3 days. Finally, the cytopathic effect (CPE) was examined and observed at a specific time interval (0–48 hr) and TCID_50_ was calculated according to Reed and Muench [79].

#### 2.7.6 Transfection

Vero E6 cells (10^5^) were seeded in 12 well plates, and the cells were microscopically investigated daily until they reached 70 % confluence. Cell cultures were transfected with 100 nM (according to the Basic siRNA Resuspension Protocol of Dharmacon, Horizon Discovery, UK) of siRNA1, siRNA2, siRNA3, siRNA4, positive siRNA, and negative siRNA according to the Lipofectamine 3000 protocol. After 24 hours, Vero E6 cells were infected with the SARS-CoV-2 virus at a multiplicity of infection (MOI) of 0.01. Infected Vero E6 cells were used as a positive control, and Vero E6 cells were used as an untreated (negative) control.

#### 2.7.7 RNA extraction and quantitative RT-PCR

Viral RNA was extracted from eight-cell cultures at specified time points using the QIAamp Viral-RNA Kit (Qiagen, Germany). cDNA was synthesized from total RNA using the High-Capacity cDNA Reverse Transcription Kit, Applied Biosystems. The quantity and quality of cDNAs were determined using a Nanodrop (NANODROP 2000c, ThermoScientific). Three monoplex real-time RT-PCR assays targeting the ORF1b and S gene regions of SARS-CoV-2 and the human GAPDH (Glyceraldehyde 3-phosphate dehydrogenase) gene as a control (reference gene) were performed. The primer sequences, presented in Table S1, were synthesized by Macrogen. A typical 20 μl reaction contained 10 μl Maxima SYBR Green qPCR Master Mix(2x), no ROX (ThermoFisher), 0.6 μl forward primer(10pmol/μl), 0.6 μl reverse primer(10pmol/μl), ROX solution 0.04 μl, 1 μL Template DNA, 7.76 μl water, nuclease-free. RT-PCR reactions were conducted using a thermal cycler (Applied Biosystem 7500 Fast) with the following conditions: 1 cycle of initial denaturation at 95°C for 10 min, followed by 40 cycles of PCR amplification (Denaturing at 95°C for 15 s, Annealing/Extending at 60°C for 60 s). We used these steps:

Step 1. Normalize to (Reference Gene): ΔCq = Cq (target gene) – Cq (reference gene)

Step 2. Exponential expression transform: ΔCq Expression = 2–ΔCq

Step 3. Average replicates and calculate the standard deviation.

Step 4. Normalize to treatment control.

Step 5. % KD = (1 – ΔΔCq) × 100.

as described in [40], [41] to analyze the data to obtain relative gene expression and the knockdown percentage.

### 2.8 Statistical analysis

All data were obtained in triplicate in at least three independent trials. The acquired data were coded, tabulated, and statistically analyzed using GraphPad Prism, version 5.01 (GraphPad Software). The data are presented as the mean standard deviation (SD). Statistical analysis was conducted using two-way ANOVA followed by the Tukey-Kramer multiple comparisons test to compare the differences between the groups. Each sample was duplicated in the qRT-PCR experiment to quantify relative gene expression using an unpaired *t*-test in GraphPad Prism, version 8.01. (GraphPad Software). The significance difference is indicated by the symbols *P ≤ 0.05, * *P ≤ 0.01, * **P ≤ 0.001, * ** *P ≤ 0.0001; ns (not significant) P > 0.05.

## 3 Results

### 3.1 In-silico design and selection of siRNAs targeting coronaviruses

This study performed whole-genome alignment on 12,649 sequences of SARS-CoV, MERS-CoV, and SARS-CoV-2 using MAFFT version 7[24]. The results revealed 10 conserved regions identified as RNAi target regions after setting a percentage identity threshold of 86.4 % using Jalview2.11[25]. This percentage was chosen to ensure conserved regions with at least 20 out of 22 nucleotides. The representative alignment (Additional file 2: Fig. S1) displayed the alignments of the 10 conserved regions identified in this analysis against the reference sequences of SARS (NC_004718.3), SARS-CoV-2 (NC_045512.2), and MERS-CoV (NC_019843.3) using Clustal Omega Clustal Omega < Multiple Sequence Alignment < EMBL-EBI. As shown in Additional file 2: Fig. S2, the ten conserved regions matched completely with different circulating variants of SARS-CoV-2 as Alpha Beta, Delta, Epsilon, Eta, Gamma, Lota, Kappa, Lambda, Zeta, Omicron BA.1, Omicron BA.2, Omicron BA.2.12.1, Omicron BA.2.75, Omicron BA.4, Omicron BA.5, Omicron BQ.1.1, Omicron EG.5.1, Omicron XBB.1.5.Table 2 shows where the conserved regions are located on the SARS-CoV-2 genome reference sequence NC_045512.2.

258 siRNAs were designed using multiple online software as i-SCORE Designer[26], OligoWalk [27], siVirus [28], Sfold [29], SCALES [30], and siPRED [31], to target the identified conserved regions as shown in Additional file 1. After applying a primary filtration based on the predicted experimental inhibition cutoff, 56 siRNAs were obtained. Their thermodynamic features such as Whole ΔG for the RNA duplex and the differential stability of the 5` and 3` ends were calculated and filtered according to [34] as the most preferable ΔG of efficient siRNA ranges between −35 and −27 kcal/mol, only 20 siRNAs passed this step.

The secondary structure of the 20 siRNAs and their minimal free energy (MFE)were examined using Sfold[29] and RNAfold[33]; only 10 siRNAs passed the second filtration step. The target accessibility of 10 siRNAs with their target regions in the mRNA of SARS-CoV-2 was assessed using IntaRNA2.0[35] and RNAup [33] as shown in Additional file 1, Table S10.

The final filtration stage involved off-target selection, where we selected the siRNAs with the fewest near-perfect matches with human mRNA transcripts in the Human genomic plus transcript (Human G+T) database was rejected using BLAST NCBI as shown in Table 3 [7]. After completing all these filtration processes, only four siRNAs were considered the most precisely targeted and effectively engineered siRNAs. Their secondary structure and minimal free energy (MFE), are presented in Additional file 2: Fig. S3, examined using Sfold[29] and RNAfold[33]. Additional file 2: Fig. S4 represents the target accessibility of four siRNAs with their target regions in the mRNA of SARS-CoV-2. The features of the final selected siRNAs used in this study, based on the previous steps, are presented in Table 1.

**Table 3:** The identity of four siRNA using BLAST NCBI against the Human genomic plus transcript (Human G+T) database.

Name	siRNA sequence(sense)*^1^	Off-target results using BLAST NCBI	Identity
siRNA 1	5`G.G.A.C.A.A.G.A.G.G.G.C.A.A.A.A.G.U.U.au3`	Homo sapiens SIX homeobox 3 (SIX3), mRNA	17/21
siRNA 2	5`G.A.C.A.A.G.A.G.G.G.C.A.A.A.A.G.U.U.A.au3`	Homo sapiens SIX homeobox 3 (SIX3), mRNA	16/21
siRNA 3	5`G.G.C.U.U.U.G.A.G.U.U.G.A.C.A.U.C.U.A.au3`	Homo sapiens trans-golgi network protein 2 (TGOLN2), transcript variant 4, mRNA	15/21
siRNA 4	5`G.U.C.U.A.A.G.G.G.U.U.U.C.U.U.U.A.A.G.au3`	No off-target	Not detected

### 3.2 siRNA cytotoxicity (CC50) in Vero E6 cells

**Fig. 3:**
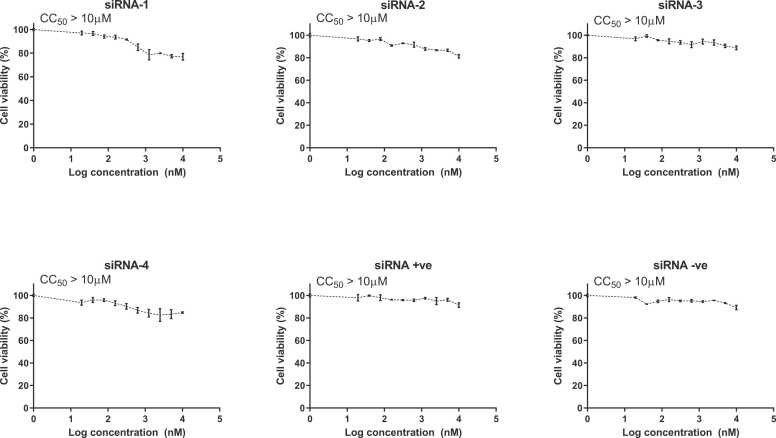
**Cytotoxicity (CC50) of siRNAs in Vero E cells** The cytotoxicity of six siRNAs (siRNA1, siRNA2, siRNA3, siRNA4, positive siRNA, negative siRNA) was tested in Vero E6 cells using a crystal violet assay. The cytotoxic effect of the designated siRNA found to be concentration-dependent. All siRNAs generated nearly identical results, with good viability at 100 nM. None of the tested siRNAs showed a cytotoxic effect on Vero E6 cells, and the CC50 for all siRNAs was better than 10 µM. The data are presented as the mean ± S.D of 3 independent replicates. GraphPad Prism (GraphPad Software, version 5.01) was used to code, tabulate, and statistically analyze the collected data.

The cytotoxicity of six siRNAs (siRNA1, siRNA2, siRNA3, siRNA4, positive siRNA, negative siRNA) was tested in Vero E6 cells using a crystal violet assay. The results showed that the cytotoxic effects of the designated siRNAs were concentration-dependent. All generated siRNAs had similar results with good viability seen at 100 nM. Fig. 3 illustrates that siRNA1, siRNA2, siRNA3, and siRNA4 at 2.5 log concentration had cell viability of 90 %, 92 %, 94 %, and 94 % respectively. At 4 log concentration, as shown in Fig. 3, none of the tested siRNAs had cytotoxic effects below 80 % on Vero E6 cells, and the CC50 for all siRNAs was greater than 10 µM.

### 3.3 Small interfering RNAs (siRNAs) reduce SARS-CoV-2 replication

**Fig. 4:**
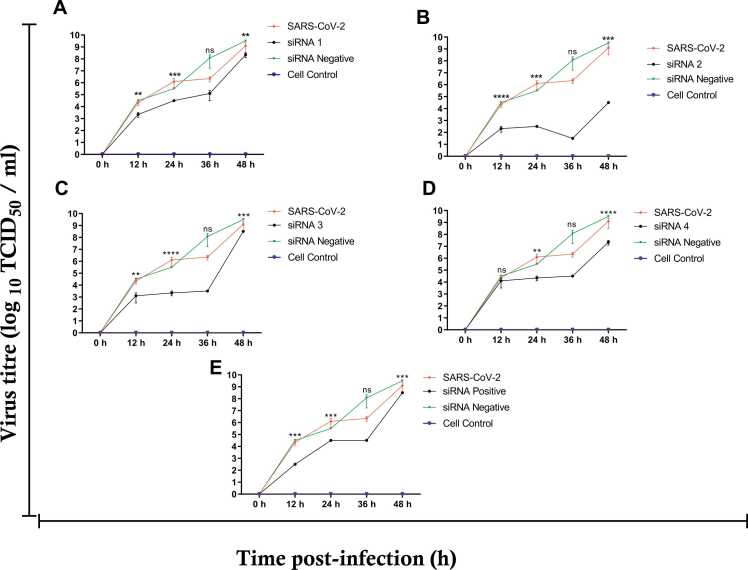
**Replication Efficiency of infected VeroE6 cells at 0, 12, 24, 36, and 48 hr post-infection using TCID50 assay at MOI 0.01.** The viral titre (log 10 TCID50 / ml) for siRNA1, siRNA2, siRNA3, siRNA4, siRNA positive, and siRNA negative at 0,12,24,36, and 48 h.p.i. is illustrated in this figure. Vero E6 cells infected with SARS-CoV-2 isolate were used as a control, and Vero E6 cells were used as an untreated control. A: siRNA1 showed the highest P value equal to 0.0001 at 24 h.p.i compared to the P value at 12, 36, and 48 h.p.i. B: When comparing the P values at 12. 24, 36, and 48 hours post-infection, siRNA2 displayed a P value of ≤ 0.0001 at 12 h.p.i. C: Compared to the P values at 12, 36, and 48 hours post-infection, siRNA3 demonstrated a significant P value of less than 0.0001 at 24 hours post-infection. D: siRNA4 showed a significant P value of ≤ 0.0001 at 48 h.p.i compared to the P value of 0.0020 at 24 h.p.i and showed no significant P value at 12 and 48 h.p.i. E: siRNA positive showed a P value ≤ 0.0001 at 12, 24, and 48 h.p.i. All data were obtained in triplicate in at least three independent trials. All results were normalized against siRNA negative. The acquired data were coded, tabulated, and statistically analyzed using GraphPad Prism, version 8.0 (GraphPad Software). The data are shown as mean standard deviation (SD). Statistical analysis was carried out using two-way ANOVA followed by the Dunnet multiple comparisons test to compare the differences between the groups. The significant difference is shown by the symbols *p ≤ 0.05, * *p ≤ 0.01, * **p ≤ 0.001, * ** *p ≤ 0.0001; ns (not significant) p > 0.05.

At 0,12,24,36, and 48 hours post-infection, the supernatant of siRNA1, siRNA2, siRNA3, siRNA4, positive siRNA, and negative siRNA was collected to measure the virus titer. All siRNAs reduced viral gene expression with varying levels of effectiveness, and all data were standardized to the negative siRNA (scrambled siRNA). At 12 h.p.i, the efficacy of the four siRNAs and their P value were highly significant as seen in Fig. 4. siRNA 2 revealed the most significant(P value≤0.0001) inhibition of viral growth among the other siRNAs with2.3 log 10 TCID50 compared to 4.5 log 10 TCID50 of negative siRNA. siRNA 2 maintained the ability to prevent viral replication at 24, 36, and 48 h.p.i with 2.5, 1.5, and 4.5 log 10 TCID50, respectively, and with P value ≤ 0.001 at 24 and 48 h.p.i. While siRNA 3 inhibited viral growth by 3.34 and 3.5 log 10 TCID50 at 24 and 36 h.p.i, respectively, and the highest P value of ≤ 0.0001 at 24 h.p.i compared to other siRNAs. In comparison to other siRNAs and the negative siRNA (scrambled siRNA), which has 9.5 log 10 TCID50, Fig. 4 shows a significant P value of ≤ 0.0001 for siRNA4 at 48 h.p.i. with 7.3 log 10 TCID50. At 12, 24, 36, and 48 h.p.i, positive siRNA displayed a P value of ≤ 0.001 compared to the negative siRNA. These findings suggest that the transfection is proceeding satisfactorily.

### 3.4 qRT-PCR quantified siRNAs antiviral inhibition activity

**Fig. 5:**
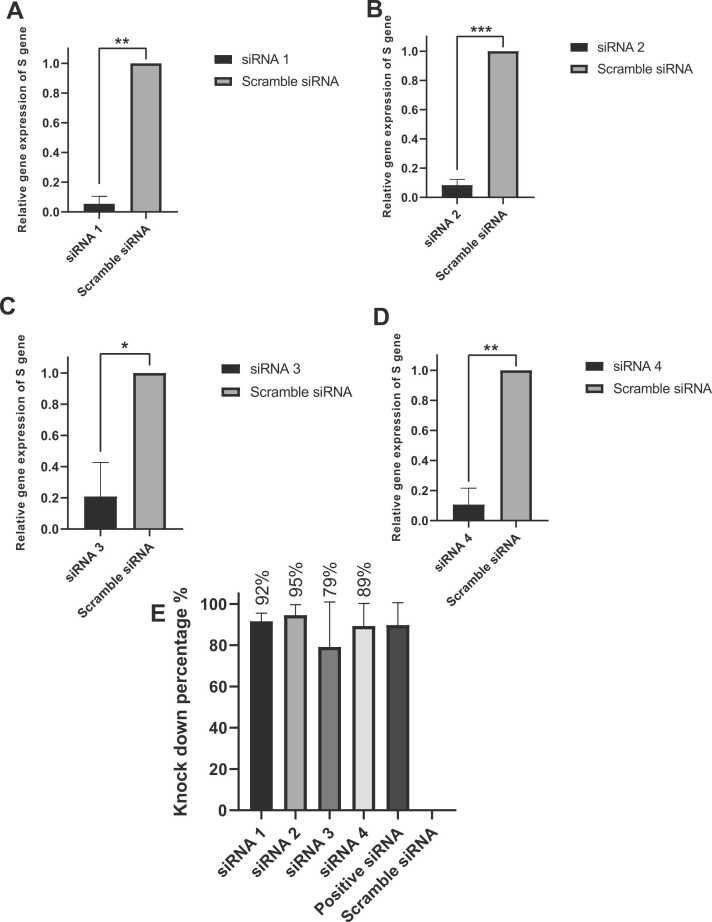
**Knockdown effect of siRNAs transfection on S gene in infected VeroE6 cells.** All siRNAs significantly decreased viral replication at 24 hours post-infection. A: siRNA1 reduced the S gene with a P value of ≤ 0.01. B: siRNA2 showed high significance in reducing SARS-CoV-2 mRNA with a P value of ≤ 0.001. C: siRNA3 had a P value of ≤ 0.05. D: siRNA4 also reduced the S gene with a P value ≤ 0.01. E: As compared to scramble siRNA, the knockdown percentages of all the siRNAs were: A:siRNA1 (92 %), B:siRNA2 (95 %), C:siRNA3 (79 %), and D:siRNA4 (89 %). All results were normalized against scramble siRNA and quantitative examination (n = 2 in each group). Means ± SEMs represent the values by GraphPad Prism, version 8. Significance was determined using an unpaired t test. The significant difference is shown by the symbols * P ≤ 0.05, * * P ≤ 0.01, * ** P ≤ 0.001, * ** * P ≤ 0.0001, and ns (not significant)) P > 0.05. Positive siRNA is utilized to ensure the transfection is going effectively.

At 12 h.p.i., Figure S5 (Additional file 2) shows that siRNA2 knocked down the virus’s S gene expression by 79 % and a significance P value of ≤ 0.01. At the same time, siRNA1 and siRNA4 reduced S gene expression with P values of ≤ 0.05 and knockdown percentages of 68 and 84, respectively. Fig. 5 illustrates that at 24 hours post-infection, all siRNAs had a significant impact on reducing S gene expression, siRNA1 (92 % knockdown) and siRNA4(89 %knockdown) showing P values of ≤ 0.01. The expression of the S gene was also decreased by siRNA2 (95 % knockdown) and siRNA3(79 %knockdown) with P values of ≤ 0.001 and ≤ 0.05, respectively. In Figure S6 (Additional file 2), siRNA2 still had the most significant impact with a P value of ≤ 0.001 and 94 knockdown percentage at 36 h.p.i. Figure S7 (Additional file 2) illustrates how the effect of siRNAs on gene silencing diminished after 48 h.p.i. However, siRNA2 continued to demonstrate a decrease in S gene expression with a P value of ≤ 0.05 and 59 % knockdown.

**Fig. 6:**
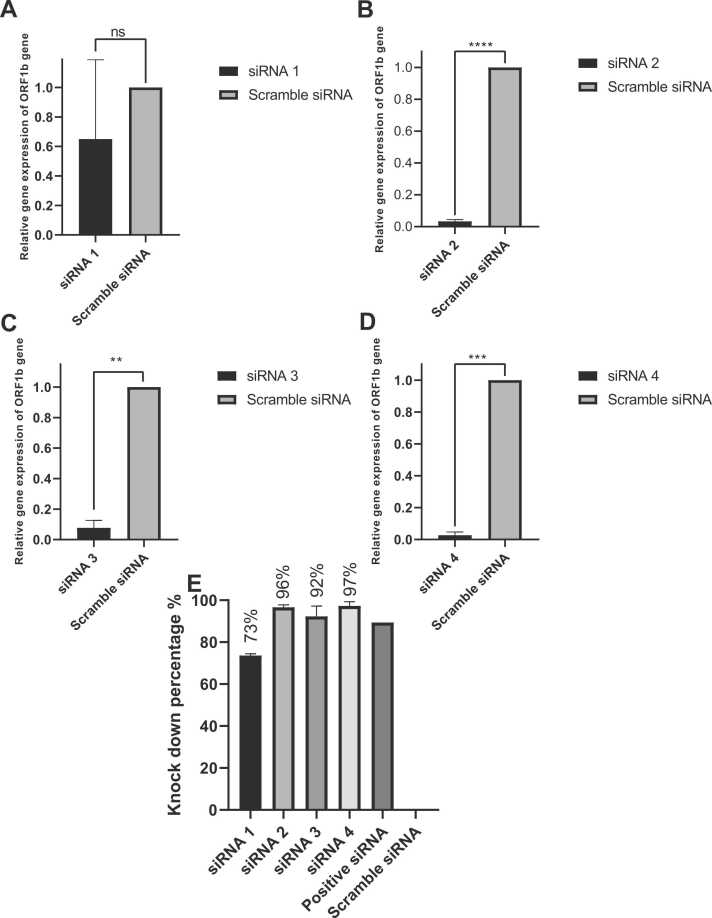
**Knockdown effect of siRNAs transfection on the ORF1b gene in infected VeroE6 cells.** At 24 h.p.i, all siRNAs significantly reduced viral replication. A: siRNA1 reduced the mRNA of the ORF1b gene, but the reduction was not statistically significant. B: siRNA2 demonstrated a P value of ≤ 0.0001 in ORF1b mRNA reduction. C: siRNA 3 reduced the mRNA of ORF1b with a P value of ≤ 0.01while D: siRNA4 had a P value of ≤ 0.001. E: Compared to scramble siRNA, the knockdown percentages of all the siRNAs were as follows: A:siRNA1 (73 %), B:siRNA2 (96 %), C:siRNA3 (92 %), and D:siRNA4 (97 %). All results were normalized against scramble siRNA, and a quantitative examination was performed (n = 2 for each group). Means ± SEMs were used to represent the values by GraphPad Prism, version 8. Significance was determined using an unpaired t test. The symbols * P ≤ 0.05, * * P ≤ 0.01, * ** P ≤ 0.001, * ** * P ≤ 0.0001, and ns (not significant)) P > 0.05 indicate significant differences. The positive siRNA was utilized to ensure effective transfection.

siRNA2 and siRNA4 showed highly significant knockdown of the virus’s ORF1b gene at 12 h.p.i with 95 % and 92 %, respectively, and a P value of ≤ 0.001compared to siRNA1 and siRNA3 as shown in Figure S8 (Additional file 2). As shown in Fig. 6 and S9 (Additional file 2), siRNA4 demonstrated a P value of ≤ 0.001at 24 h.p.i. and a P value of < ≤0.01at 36 h.p.i.but siRNA2 showed great significance in lowering ORF1b expression with a P value of ≤ 0.0001at24 and 36 h.p.i. siRNA3 showed a significant P value of ≤ 0.01at 24 and 12 h.p.i as shown in Fig. 6 and Figure S8 (Additional file 2) respectively. Even though the impact of the siRNAs decreased at 48 h.p.i, siRNA2 and siRNA4 still showed a significant reduction in the expression of ORF1b with a P value of ≤ 0.05and 49 and 54knockdown percentages, respectively as shown in Figure S10 (Additional file 2).

## 4 Discussion

To date, approximately seven million people have died as a result of the COVID-19 pandemic, which was caused by a positive-sense RNA virus known as SARS-CoV-2. Traditional vaccine development takes 6–8 months; during this time, the virus undergoes several mutations in the candidate protein chosen for vaccine development[42]. By the time the protein-based vaccine is available, the virus will have undergone several mutations, and antibodies against the viral sequence may no longer effectively limit the newly mutated viruses[43]. Genetic variance analyses of the entire genome in 48,635 SARS-CoV-2 samples, compared to the reference genome (Wuhan genome) NC 045512.2, revealed a reasonable average of 7.23 mutations per sample[44]. The SARS-CoV-2 genome's proclivity for adaptive mutations may have made it extremely pathogenic, complicating drug and vaccine development[6], [45]. SARS-CoV-2 genetic variations, even within the same country, pose a challenge to finding a universally applicable therapeutic agent[46], [47]. Treatment challenges necessitate a new dimension, especially when an effective antiviral agent is needed. Several drugs used to treat SARS-CoV and MERS-CoV were discovered to be ineffective against SARS-CoV-2[48]. Due to the lack of SARS-CoV-2 specific drugs, we are looking for an effective and specific therapeutic approach. RNAi(RNA interference) is a gene-silencing mechanism that can be activated by siRNA[49] and has the potential to prevent pathogenic viral replication and infection in animal cells[50]. HCV[51], Influenza[52] and HIV were restricted using siRNA-silencing technology[53]. Recent research shows that siRNAs can suppress gene expression and prevent SARS-Cov and MERS-CoVviral replication in cultured cells[54], [55], [56], [57].

As SARS-CoV-2 adapts to the host, it undergoes significant genetic alterations[58]. Single nucleotide polymorphisms (SNPs) and deletions, primarily in the spike (S) protein, result from SARS-CoV-2's ongoing evolution and genomic mutation. These changes could lead to increased transmissibility or infectivity, evasion of the immune system through ineffective memory responses or reduced sensitivity to current vaccines, or be missed by traditional diagnostic methods[59]. This may pave the way for precision/personalized medicine in treating SARS-CoV-2 patients. The conserved and diverse potential targets in the reference sequences of the SARS-CoV-2 genome serve as a basis for developing specific siRNA-based treatments.

RNAi technology can potentially inhibit SARS-CoV-2 viral replication by targeting evolving viral sequences, including those resulting from mutations. [60]. The SARS-CoV-2 genome comprises 14 open reading frames (ORFs) and 27 proteins[55]. ORF1a and ORF1b have highly conserved sequences in the annotated genomes of SARS-CoV-2 and previous beta coronaviruses like SARS-CoV and MERS-CoV[61]. The reference sequences of the SARS-CoV-2 genome provide a variety of conserved potential targets that motivate us to investigate siRNA-based therapies.

Our use of an integrative bioinformatics methodology in this study, as illustrated in Fig. 2, distinguished us from earlier research conducted by Bowden-Reid et al.[62] and Houbron, É et al.[63]. We were able to identify four siRNAs that are highly specific and well-designed. These siRNAs target specific conserved regions in the SARS-CoV-2 genome. These regions encode non-structural proteins (NSPs) 8,12 and 14. Many crucial enzymes for RNA processing and viral replication, such as RNA-dependent RNA polymerase (RdRp) and N7-guanine methyltransferase(MTase)[64] are encoded by these NSPs. NSP8 is critical in extending the template RNA-binding surface as a cofactor with NSP7 to bind to NSP12 as a viral polymerase complex. NSP8 also acts as an innate immune suppressor, facilitating viral replication and transcription[65]. NSP12, also known as the viral RdRp, possesses the catalytic activity necessary for viral replication. NSP12 requires the cofactors NSP7 and NSP8 to synthesize RNA as it has minimal activity. NSP7 and NSP8 function as primases, stabilizing the RNA-binding region of NSP12 for SARS-CoV-2 genome replication. SARS-CoV and SARS-CoV-2 NSP12 share 96–98 percent identity, suggesting their structure and function are likely identical[66]. NSP14 is a highly conserved NSP known for its 3′ to 5′ ExoN activity and guanine -N7-MTase activity, which mediates RNA capping in collaboration with NSP12, NSP13, and NSP16. NSP14 is an S-adenosylmethionine (SAM)-dependent MTase that utilizes SAM as a methyl donor, which is needed for viral replication[67].

In our in vitro studies, we assessed the cellular toxicity of each designed siRNA in the Vero E6 cell line before inducing virus infection and conducting qPCR. The results indicated that the tested siRNAs did not exhibit any cytotoxic effects on the Vero E6 cell line at a concentration of 100 nM. We also performed the TCID50 assay to assess the reduction in viral load. The four siRNAs decreased the viral load at 12 h.p.i. while the effect of siRNA2 and siRNA4 on decreasing viral load remained until 48 h.p.i.

At 24 h.p.i, siRNA2 knocked down the virus’s S gene by 95 % compared to the negative siRNA (scrambled), demonstrating a significant inhibitory effect on viral replication.

This reduction in viral replication can be attributed to the decreased production of NSP8 (Primase), which acts as a cofactor for NSP12. NSP12 and NSP8 are crucial for forming the entire RNA polymerase replicative machinery[68]. At 36 h.p.i, siRNA3 exhibited a substantial decrease in viral growth by 79 % due to it is effect in silencing NSP14, which is essential for viral replication and transcription. CoV NSP14 is necessary for viral replication and transcription. In the case of SARS-CoV-2, the ExoN domain of NSP14 functions as a proofreader, preventing lethal mutagenesis, whereas the C-terminal domain is a methyltransferase for mRNA capping. In addition to high-fidelity replication, ExoN is considered important in RNA synthesis, resistance to antiviral nucleoside analogs, fitness, immune antagonism, virulence, and promoting recombination for virus evolution[68]. During the 2020 pandemic, NSP14 exhibited minimal mutational variation. Only M501 and N129 show mutational rates above 0.01[69]. siRNA4 also demonstrated a significant reduction in viral growth at 12,24,36, and 48 h.p.i, with reductions of 92 %,97 %, 78 %, and 54 % respectively. This effectiveness could be due to the silencing of NSP12, an RNA-dependent RNA polymerase (RdRp), and forms a viral replication complex with NSP7, NSP8, and other essential components of the RNA synthesis machinery[65]. qRT-PCR testing, a precise technique for detecting and quantifying messenger RNA, was utilized to detect SARS-CoV-2 in biological specimens. The amount of viral RNA in the sample correlates inversely with the cycle threshold (Ct) value. We targeted structural genes like the spike (S)gene and species-specific accessory genes that aid in viral replication, such as the open reading frame 1b (ORF1b) gene, according to the Centers for Disease Control and Prevention (CDC) and World Health Organization (WHO) guidelines[70], [71], [72]. siRNA2 significantly reduced viral replication by 95 % at 24 h.p.i by targeting the NSP8 and disrupting the viral NSP7-NSP8-NSP12 holo-RdRp RTC(replication–transcription complex)[73]. siRNA3 achieved a 79 % knockdown at 36 h.p.i in the NSP 14 gene, leading to a disruption in the formation of the RNA proofreading complex that will help host proteases to degrade the viral genome and activate the immune system to fight the viral infection[74]. siRNA4 effectively reduced the transcription of the NSP12 gene by 97 % at 24 h.p.i. NSP12 is an essential enzyme in the virus life cycle, and coronavirus RTC relies on NSP12, not only for viral genome replication but also for sgRNA transcription[73].

Our workflow for designing siRNAs is comprehensive and systematic, incorporating multiple filtration stages and utilizing various integrative bioinformatics protocols and online software tools. We have developed a rigorous and specific methodology for selecting effective siRNAs while minimizing off-target effects, contributing to the development of more potent antiviral strategies, comparison to previous studies conducted by Hasan et al [80], Pandey and Verma [19], Niktab et al. [81], Chowdhury et al [21], Panda et al [82], Ayyagari [83]. These studies used an in silico approach and only one online software for designing siRNAs targeting different sites of the virus, such as ORF 1ab, S-protein, M-protein, E-protein, and RdRp[7]. The evidence supporting the potential therapeutic utility of these siRNAs in fighting viral infections is strengthened by the in vitro testing of the siRNAs created using our thorough design methodology as well as the investigations conducted by Friedrich et al [84], Ambike et al. [9], Sartaj Sohrab, S., et al. [85], and Chang et al. [86]. The experimental validations of Hariharan VN et al. [75] and Chang et al.[76] significantly contribute to the progression of siRNA-based therapeutics from preclinical research to clinical applications.[62]As mentioned in Neumeier J[77], chemical alteration occurs within the seed region of siRNA guide strands at position 2 from the 5 end, or by mixing several siRNAs, we will employ various ways in our future study to lessen the off-target effect.

In our future work, we will strengthen our study weakness by testing the combination of the four siRNAs on different SARS-CoV-2 variants. We will incorporate modified oligonucleotides for in-vivo experiments that can potentially enhance the efficacy, specificity, and safety of siRNA-based therapies for combating viral infection.

## 5 Conclusion

Although many breakthroughs have occurred in the treatment of COVID-19[78]., there still isn't any 100 % effective vaccine or drug. The approval of several siRNA-based therapeutics by the FDA is a massive breakthrough for winning against these diseases, and it was achieved only after facing multiple obstacles and challenges intrinsic to their development. We conducted our research using a very stringent computational methodology and in-vitro experimental evaluations, encompassing cytotoxicity and qPCR analysis. These efforts have singled out siRNA 2 and siRNA 4 as promising candidates for SARS-CoV-2 therapy. These seem to be new therapies that must be further pursued and developed based on overall positive profiles generated in early studies.

## Abbreviation

COVID-19:Coronavirus Disease 2019

siRNA: Small interfering RNA

RNAi: RNA interference

dsRNAs:double-stranded RNAs

SARS-CoV:Severe Acute Respiratory Syndrome

MERS-CoV: Middle East Respiratory Syndrome

HCoVs:human pathogenic coronaviruses

CoVs:Coronaviruses

ORF: open reading frame

## Ethics approval and consent to participate

Not applicable.

## Consent for publication

No details on individuals are reported within the manuscript.

Availability of data and material: The data supporting this study's findings are available upon reasonable request to the corresponding author.

## Authors' contributions

N.S: conceived the work, conducted the in-silico design of siRNAs and RT-PCR experiments, wrote the manuscript, created the figures, and shared the main idea.

S.H.M: performed the experiments related to the SARS-CoV-2 virus and revised the manuscript.

M.E: put the main idea, conceived the work, revised the manuscript, and supervised the work.

M.K, M.B, M.S: supervised the work.

All authors have read and approved the final manuscript.

## Funding

This research was funded by the 10.13039/100007787National Research Centre, Internal project number 12020108, and the 10.13039/501100002349Academy of Scientific Research and Technology, Grant ID: PRISM 5202, Egypt.

## Author Statement

We, the undersigned, declare that this manuscript is original, has not been published before, and is not currently being considered for publication elsewhere.

We confirm that the manuscript has been read and approved by all named authors and that there are no other persons who satisfied the criteria for authorship but are not listed.

We further confirm that the order of authors listed in the manuscript has been approved by all of us.

We understand that the Corresponding Author is the sole contact for the Editorial process. He/she is responsible for communicating with the other authors about progress, submissions of revisions, and final approval of proofs Signed by all authors as follows:

## CRediT authorship contribution statement

**Badawy Mohamed:** Supervision. **Shehata Mahmoud:** Supervision, Funding acquisition. **ElHefnawi Mahmoud:** Writing – review & editing, Visualization, Supervision, Resources, Project administration, Methodology, Funding acquisition, Data curation, Conceptualization. **Taibe Noha:** Writing – review & editing, Writing – original draft, Visualization, Validation, Methodology, Formal analysis, Data curation, Conceptualization. **Mahmoud Sara:** Writing – review & editing, Visualization, Validation, Resources, Methodology. **Kord Maimona:** Supervision.

## Declaration of Competing Interest

The authors declare the following financial interests (e.g., any funding for the research project)/personal relationships (e.g., the author is an employee of a profitable company) which may be considered as potential competing interests: Mahmoud ElHefnawi reports financial support was provided by National Research Centre and the Academy of Scientific Research and Technology. Mahmoud ElHefnawi has US provisional patent (Application number:63444249) licensed to N.S, S.H.M, M.E. If there are other authors, they declare that they have no known competing financial interests or personal relationships that could have appeared to influence the work reported in this paper
