# Supplementary material for: In Silico and In Vitro development of novel small interfering RNAs (siRNAs) to inhibit SARS-CoV-2
Source: Comput Struct Biotechnol J. 2025 Mar 23;27:1460–71. doi: 10.1016/j.csbj.2025.03.034 (PMC12008453; doi:10.1016/j.csbj.2025.03.034)
Supplement: Supplementary file 2 — Supplementary material [file mmc2.docx]

**Additional file**

**Title: In Silico and In Vitro Development of novel small interfering RNAs (siRNAs) to inhibit SARS -CoV-2**

**Authors: Noha Samir Taibe ^a^, Sara H. Mahmoud^b^, Maimona A.Kord^c^, Mohamed Ahmed Badawy^d^ , Mahmoud Shehata^b^, Mahmoud Elhefnawi^e^***

**Additional file 2: Figures**
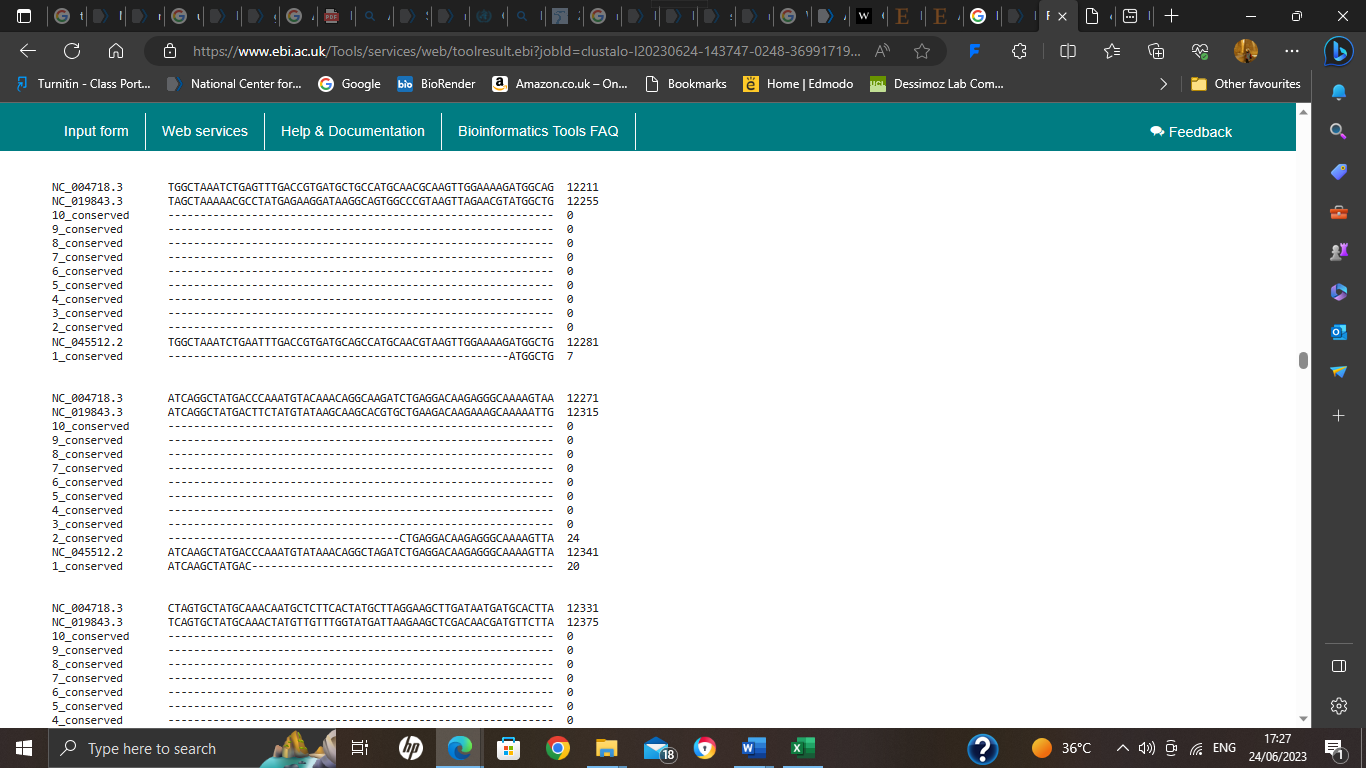


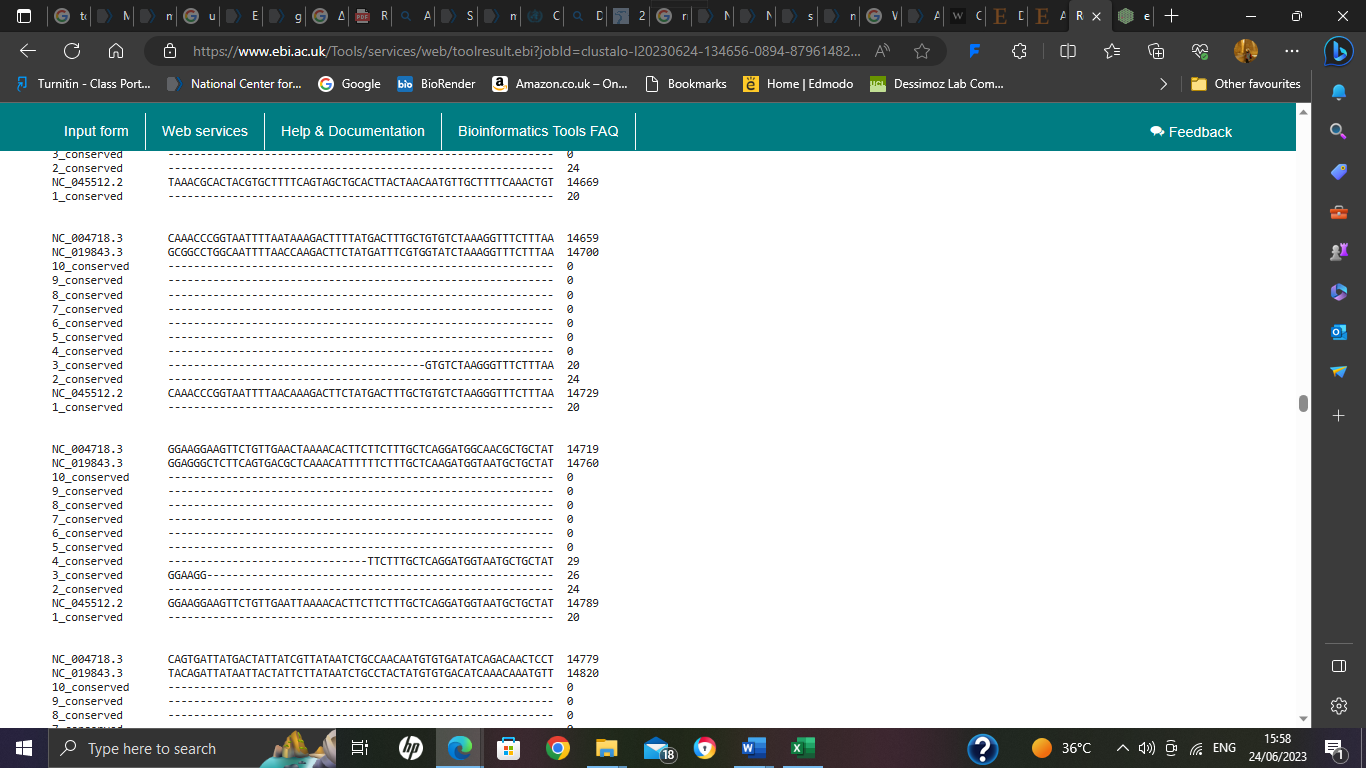


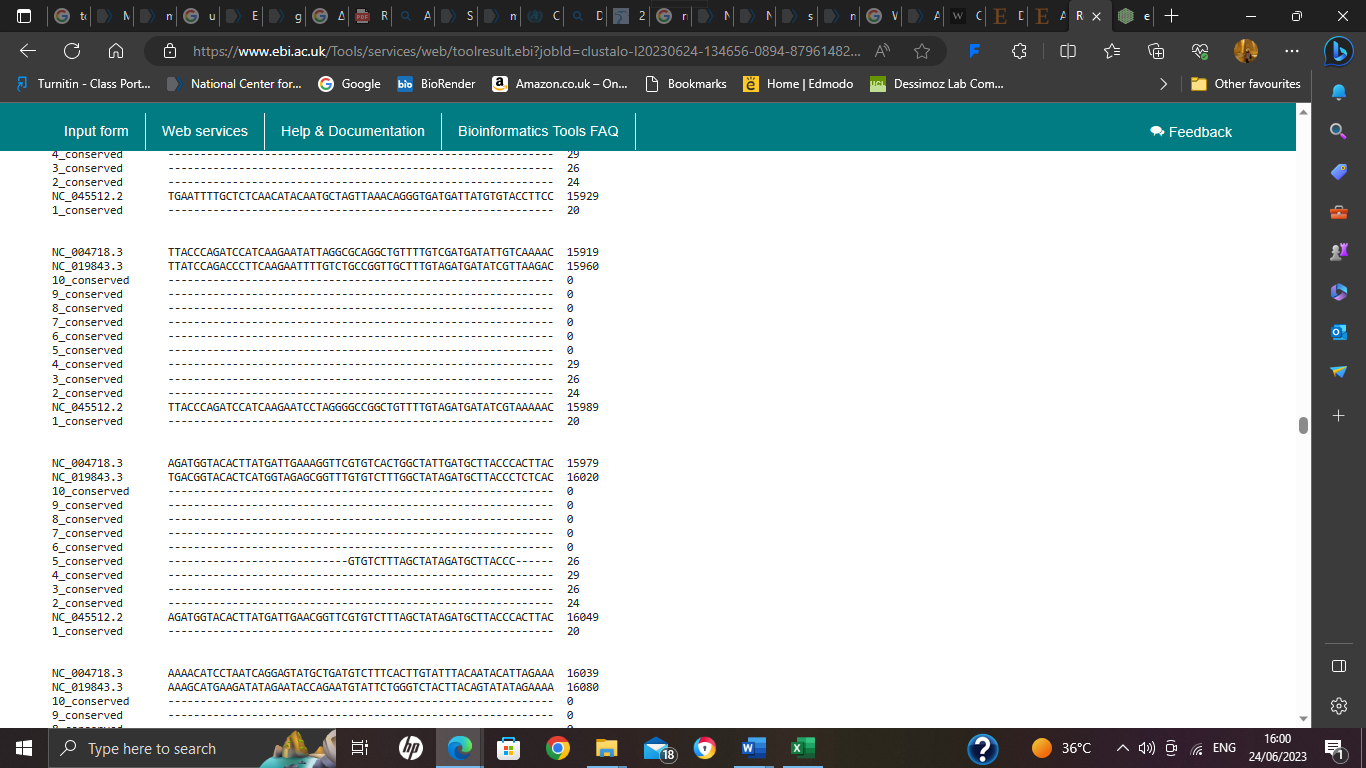


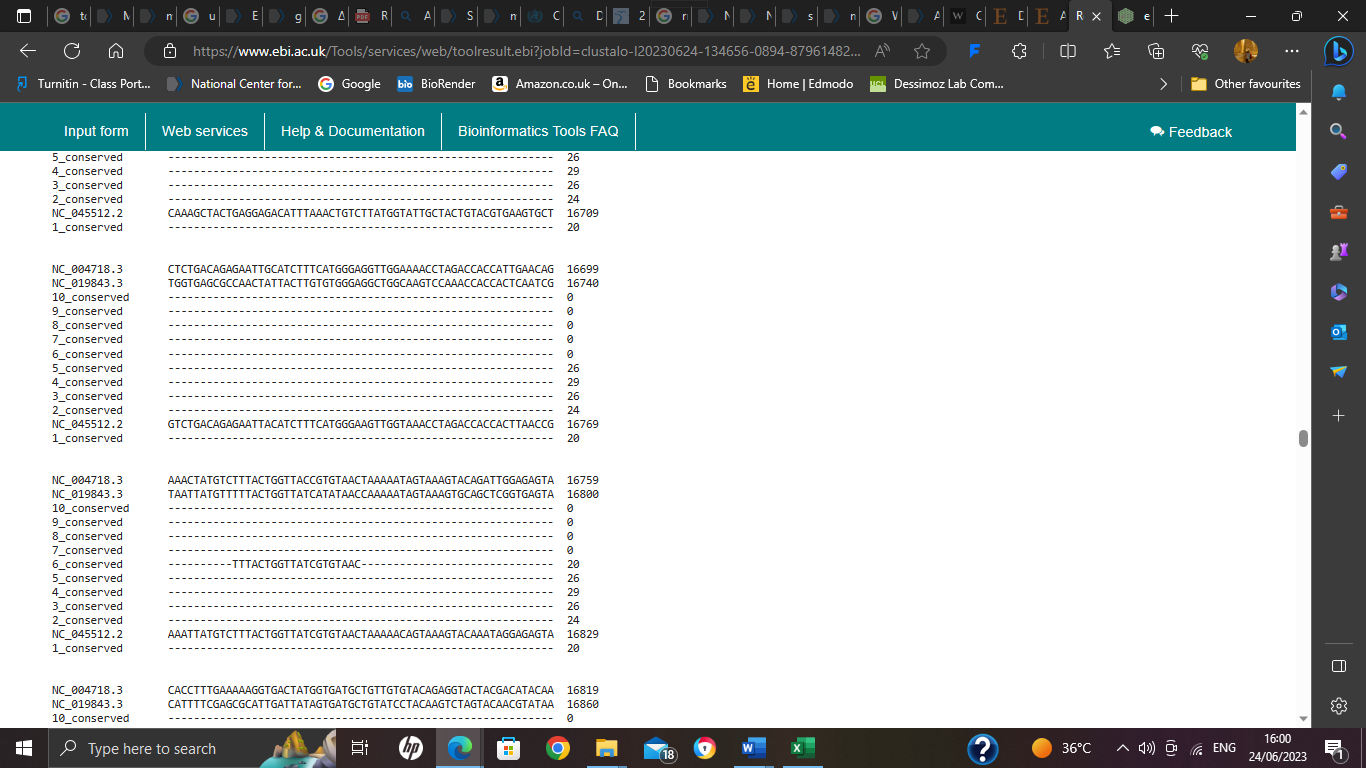


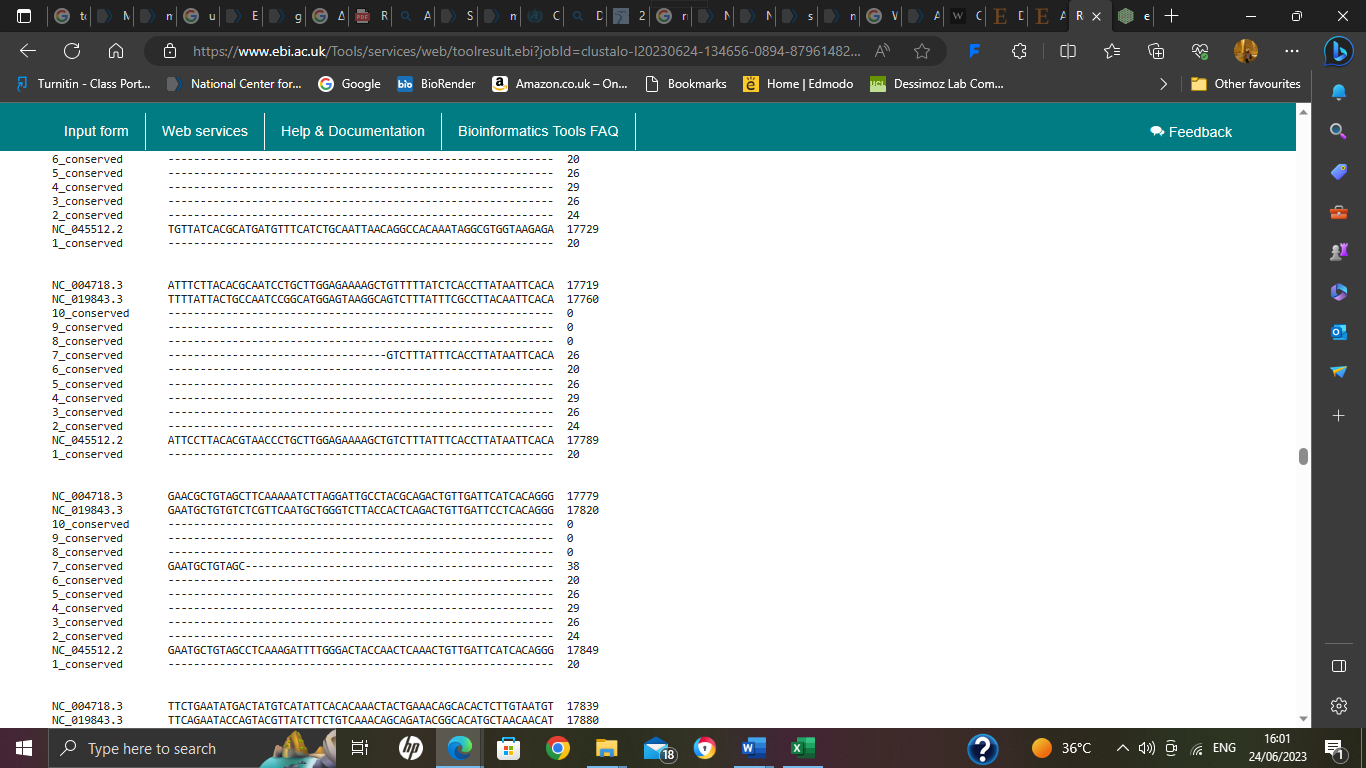


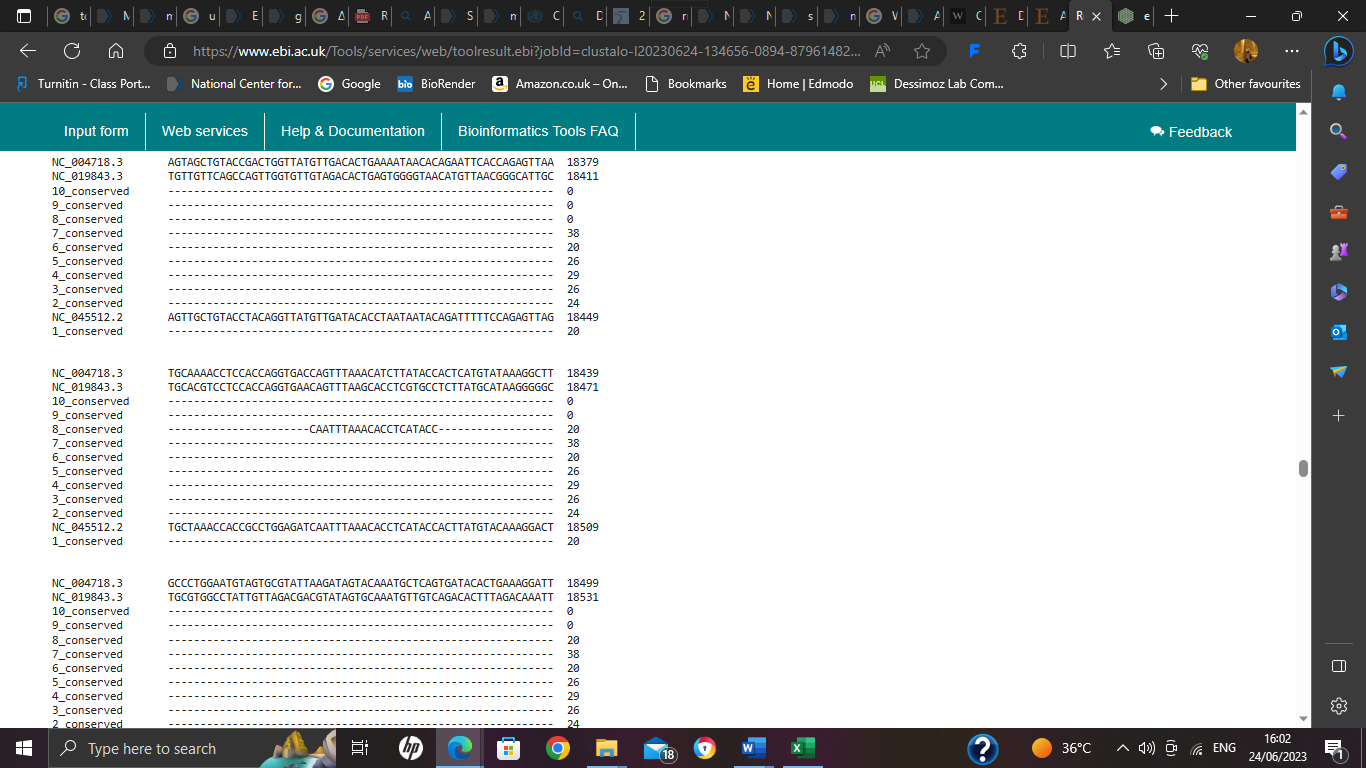


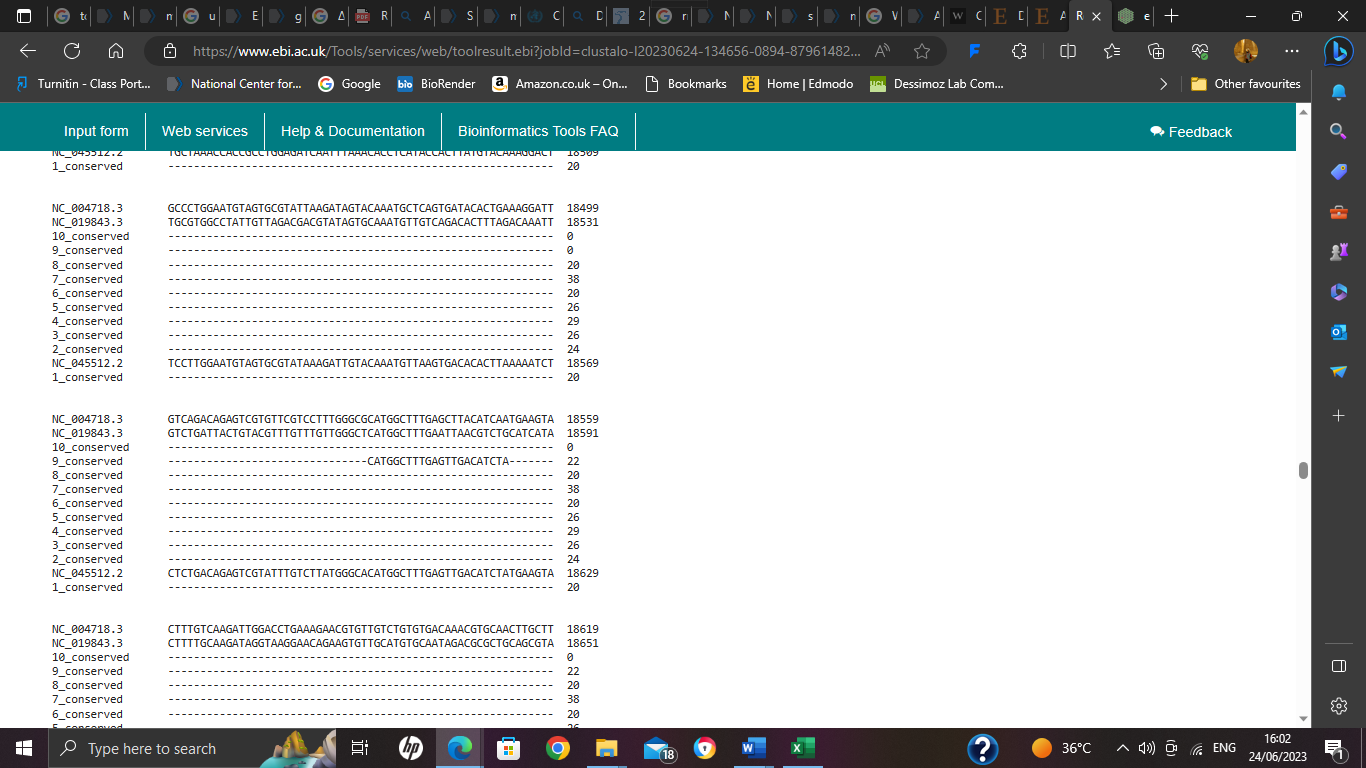


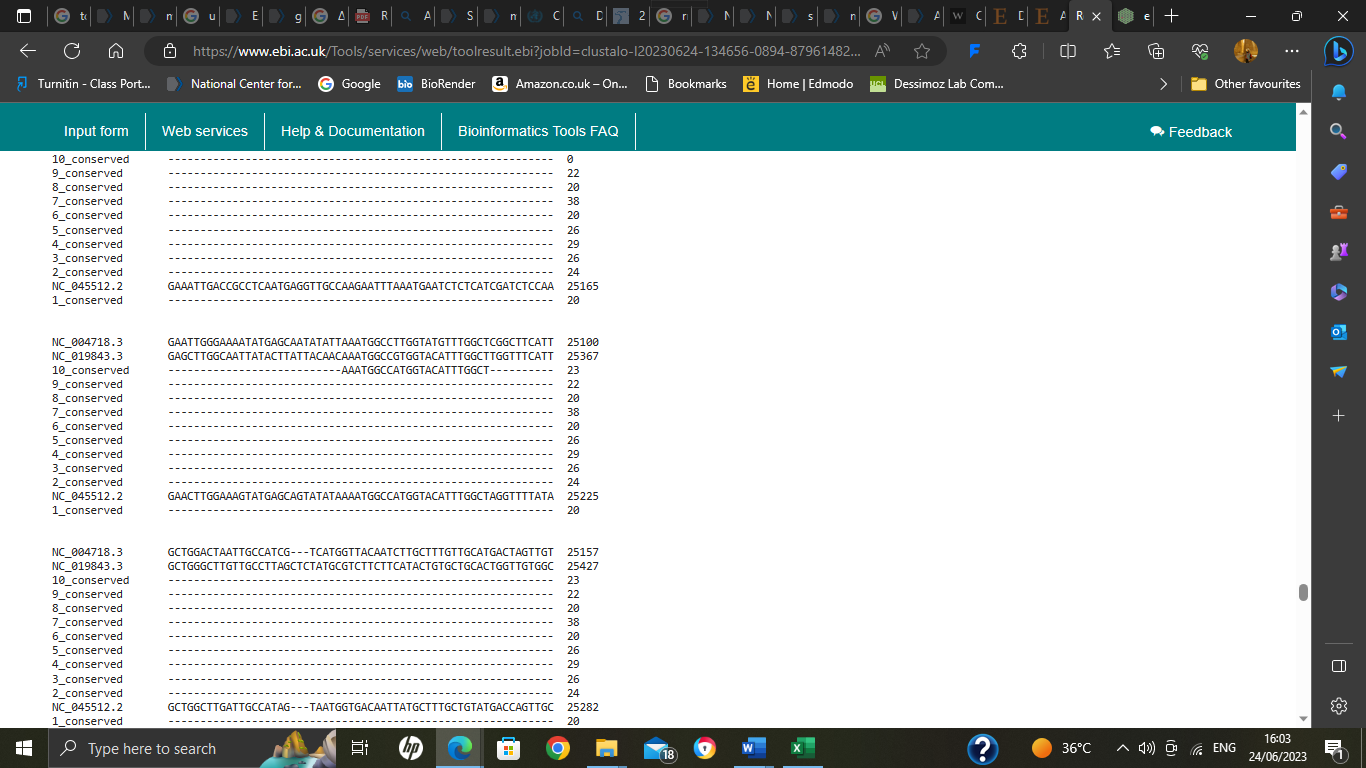


**Additional file 2: Fig. S1: Alignment of 10 conserved regions against SARS, MERS, and SARS-CoV-2 reference sequences.**

This figure displays the outcome of an alignment using Clustal Omega of 10 conserved regions against the reference sequences for SARS (NC 004718.3), SARS-CoV-2 (NC 045512.2), and MERS (NC 019843.3).


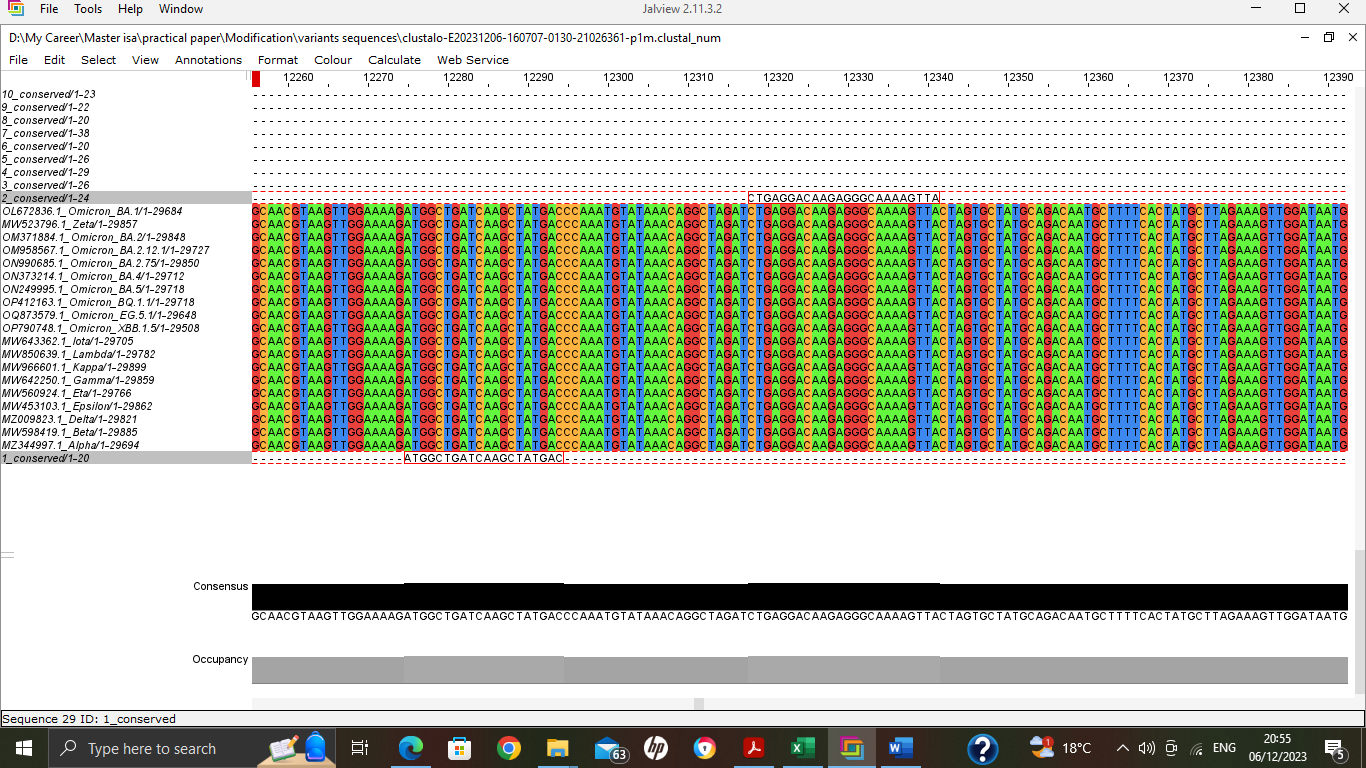

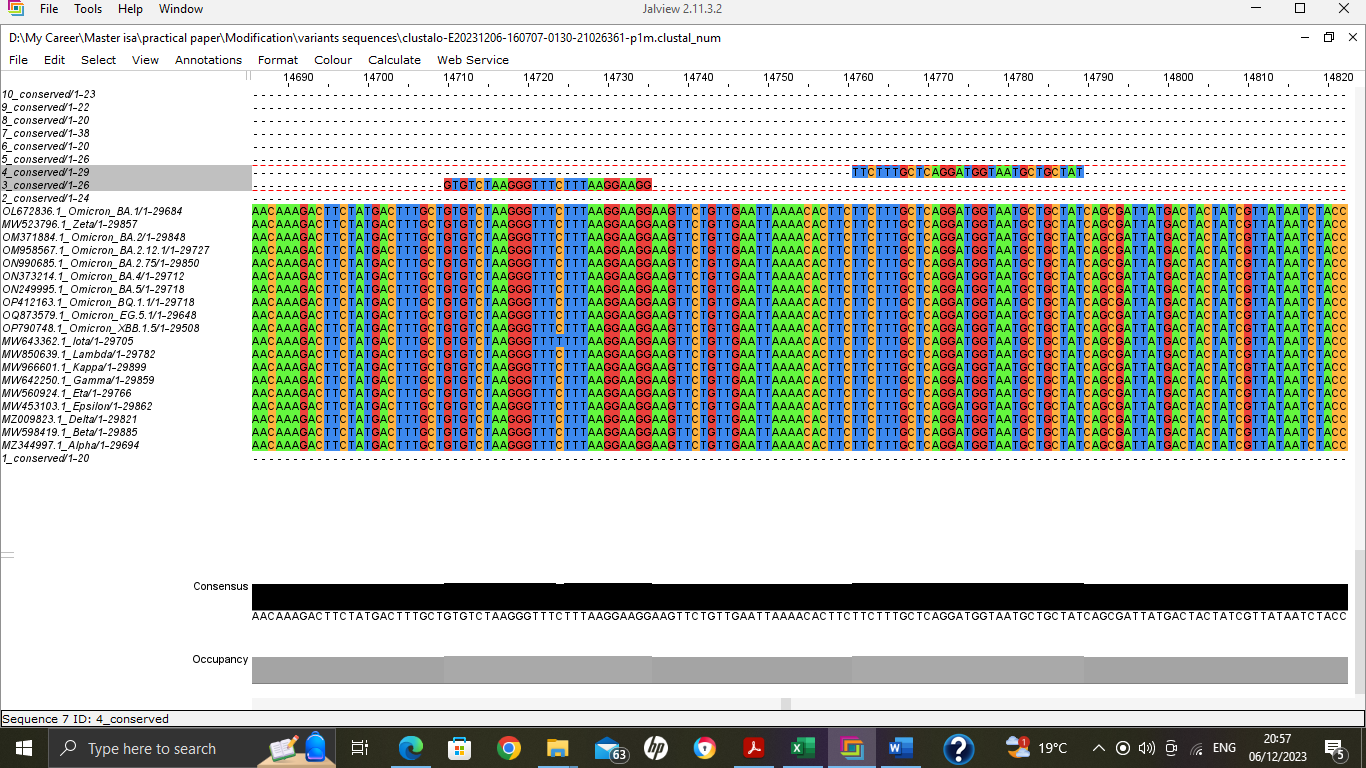

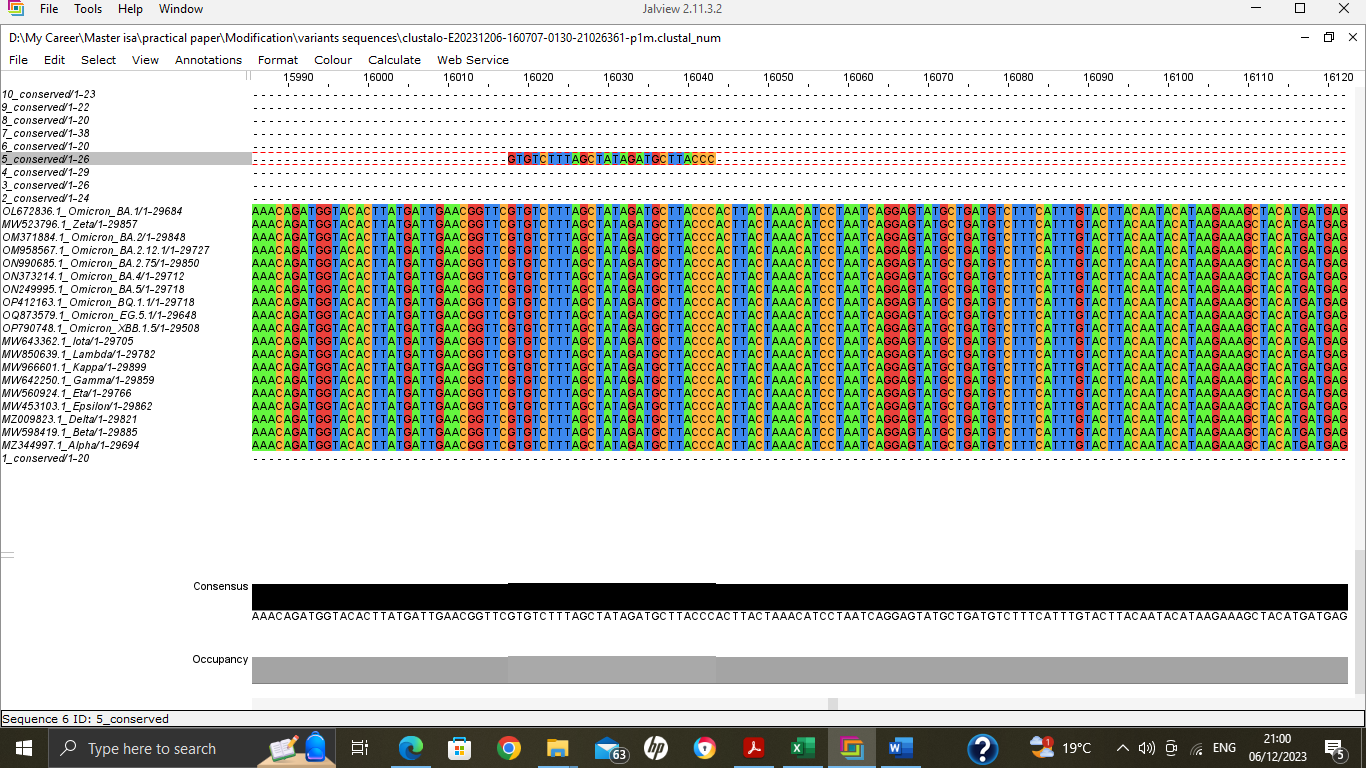

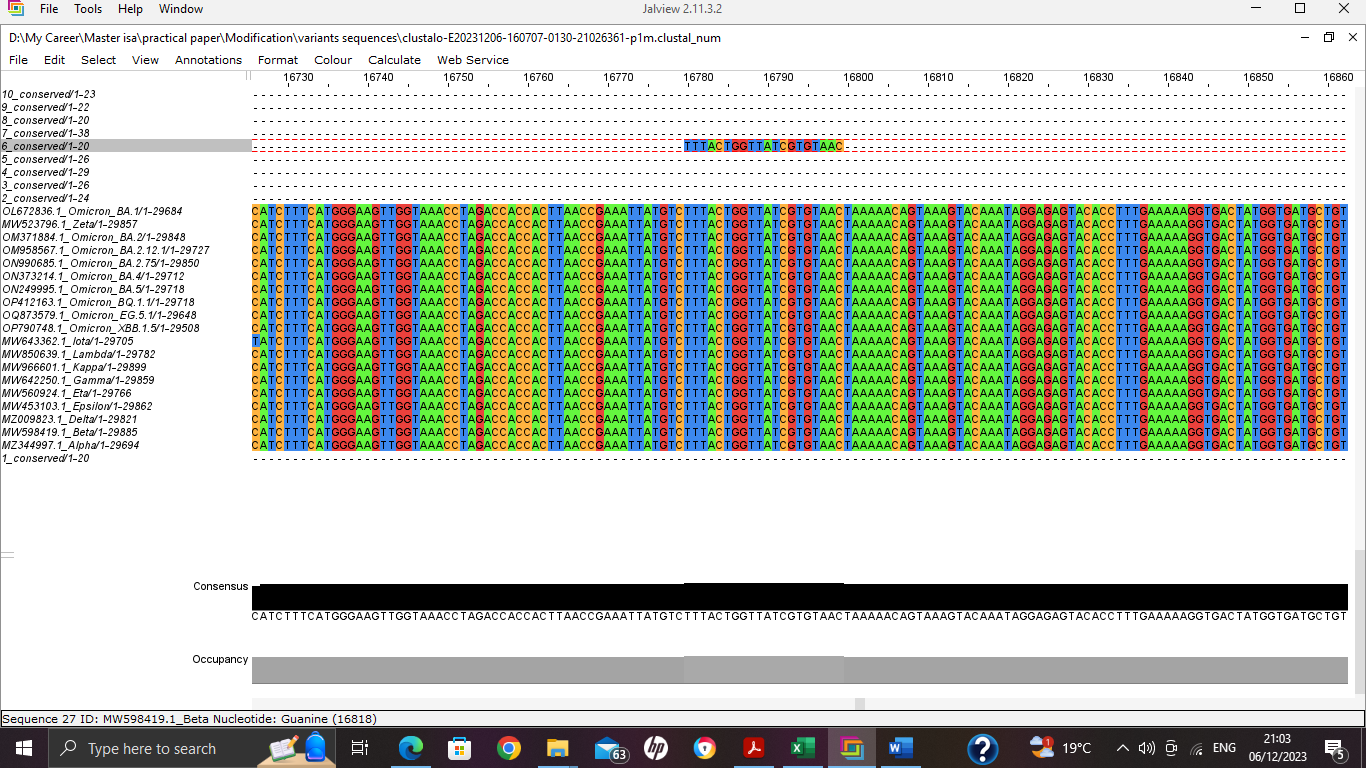

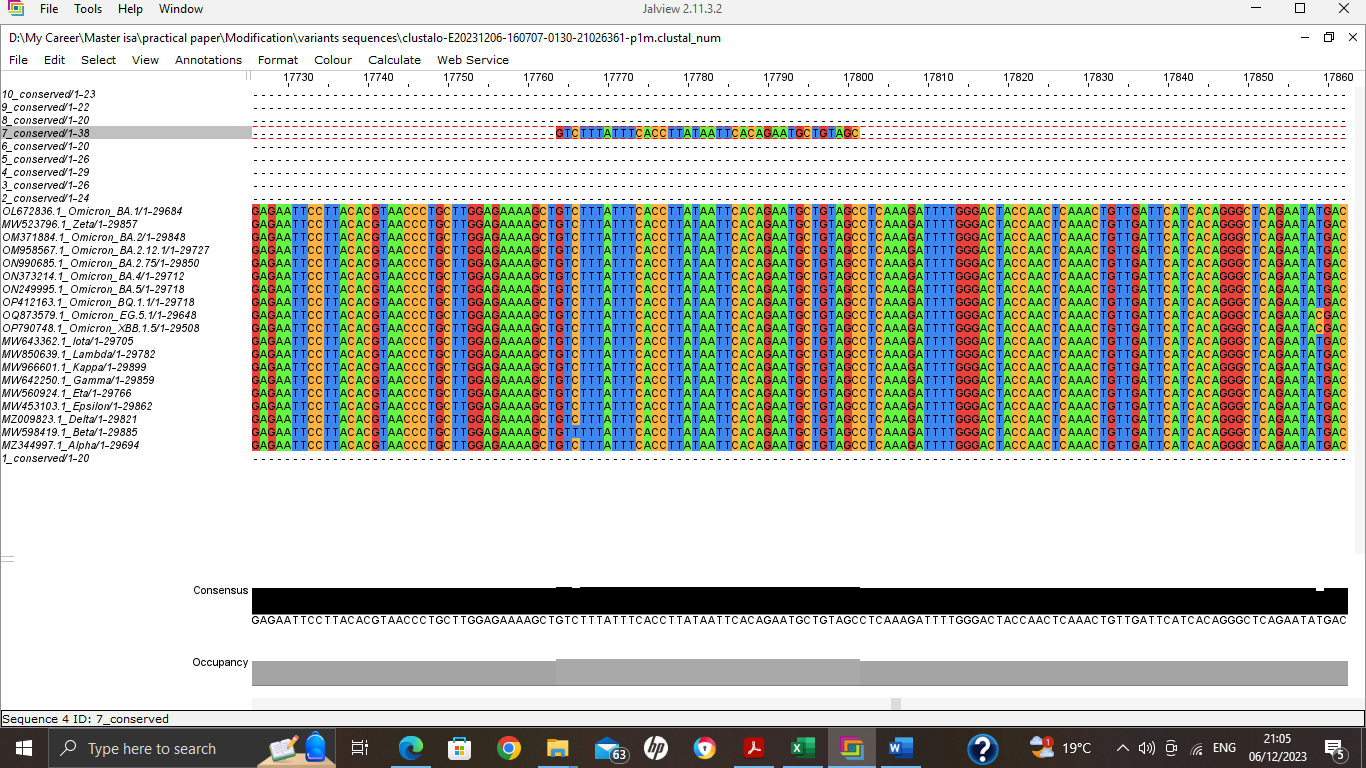

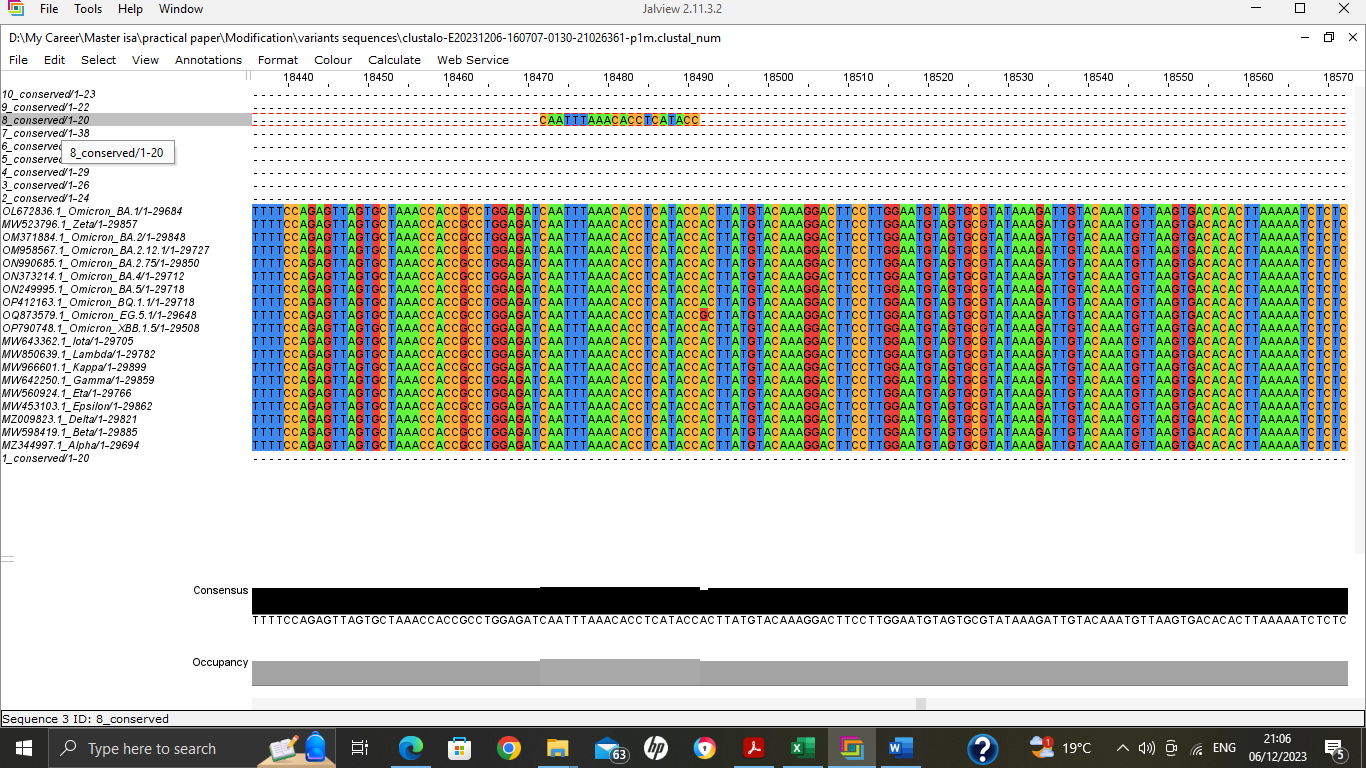

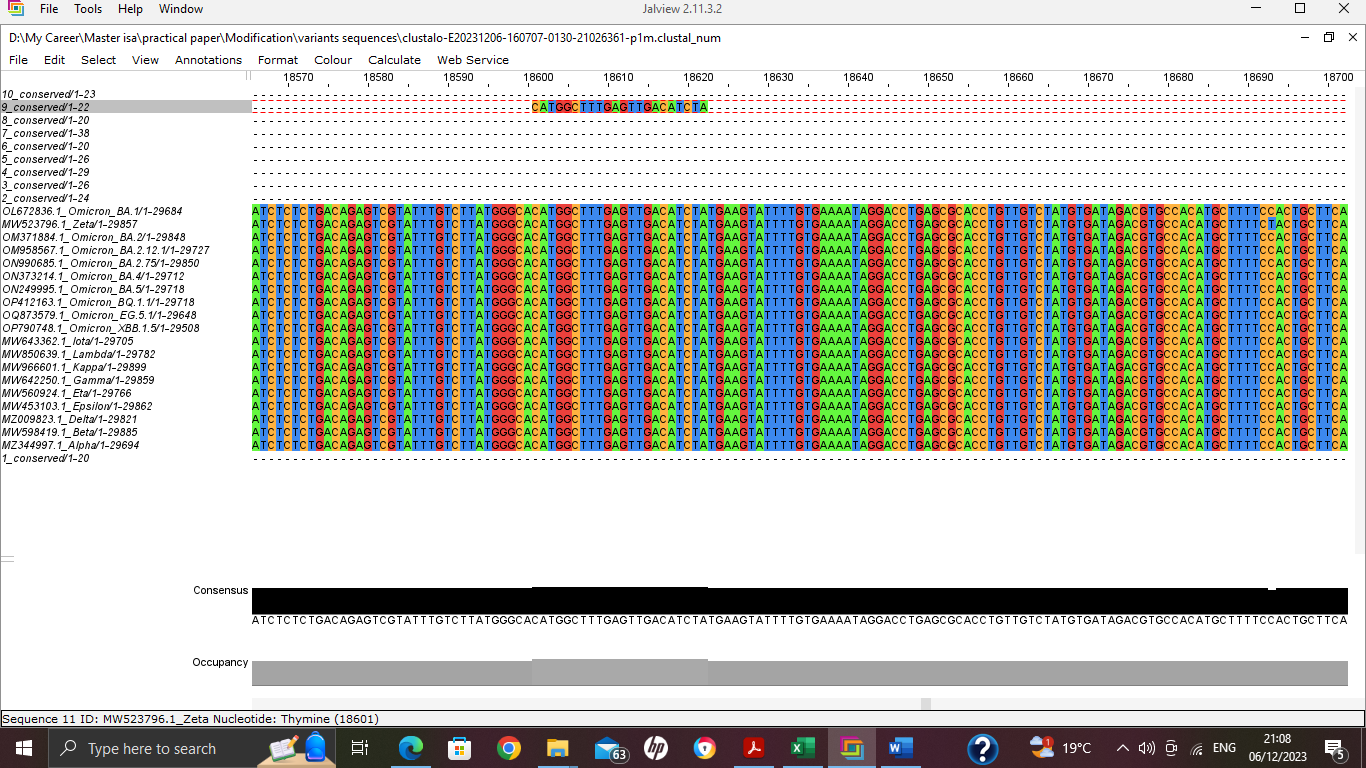

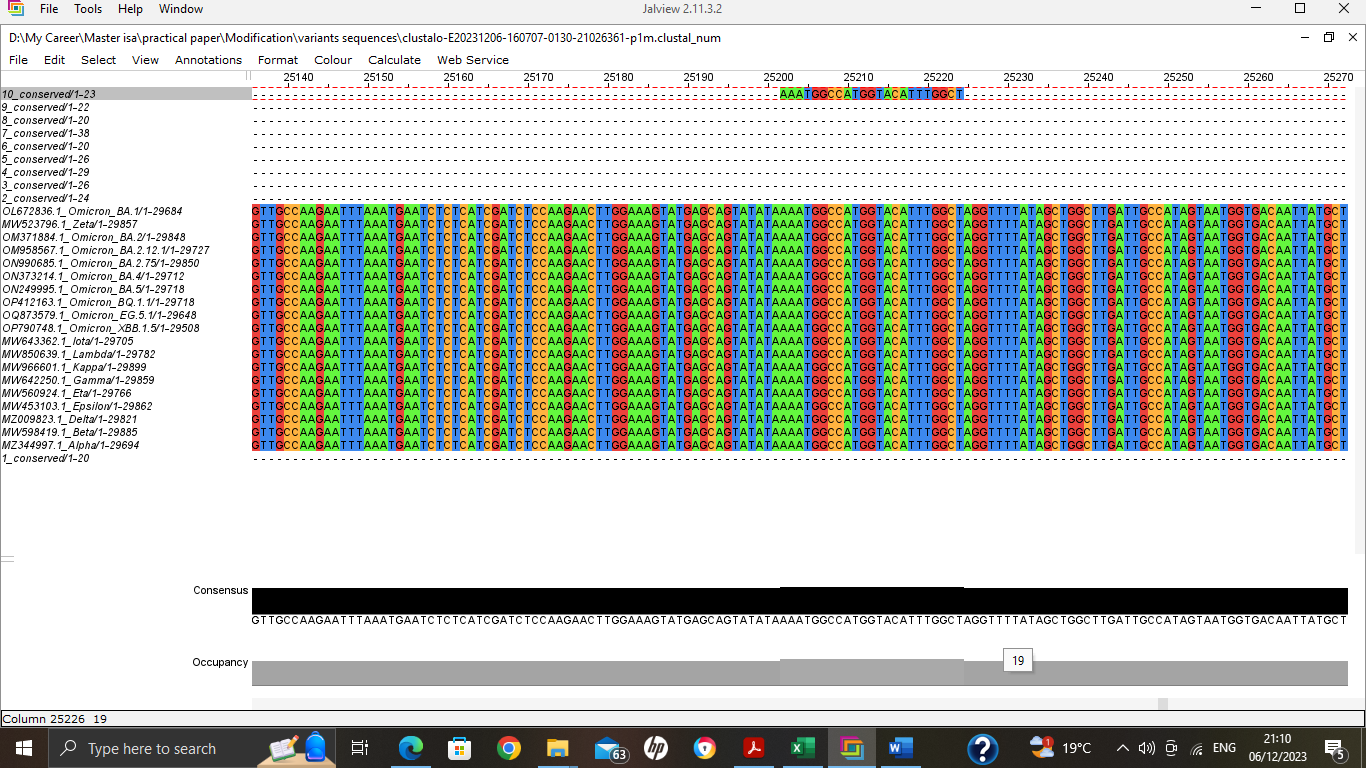


**Additional file 2: Fig. S2: Alignment of 10 conserved regions against different circulating variants of SARS-CoV-2.**

**Ten conserved regions are aligned against several SARS-CoV-2 variants that are currently circulating such as Alpha (B.1.1.7, 20I/501Y.V1, VOC 202012/01), Beta(B.1.351, 20H/501Y.V2), Delta (B.1.617.2, 21J), Epsilon(B.1.427, California(CA)), Eta(B.1.525, 20A/S:484K), Gamma(P.1, B.1.1.28.1,), Lota (P.1, B.1.1.28.1,), Kappa(B.1.617.1, 20A/S:154K), Lambda(C.37, B.1.1.1.C37), Zeta(P.2, B.1.1.28.2), Omicron BA.1(BA.1 (previously B.1.1.529)), Omicron BA.2(BA.2) Omicron BA.2.12.1, Omicron BA.2.75, Omicron BA.4, Omicron BA.5, Omicron BQ.1.1, Omicron EG.5.1, Omicron XBB.1.5 according to latest update 4/September/2023 (SARS-CoV-2 variants ~ ViralZone (expasy.org)). The results of alignments indicate a complete match of the 10 conserved regions that result from multiple sequence alignment of stage 2.**


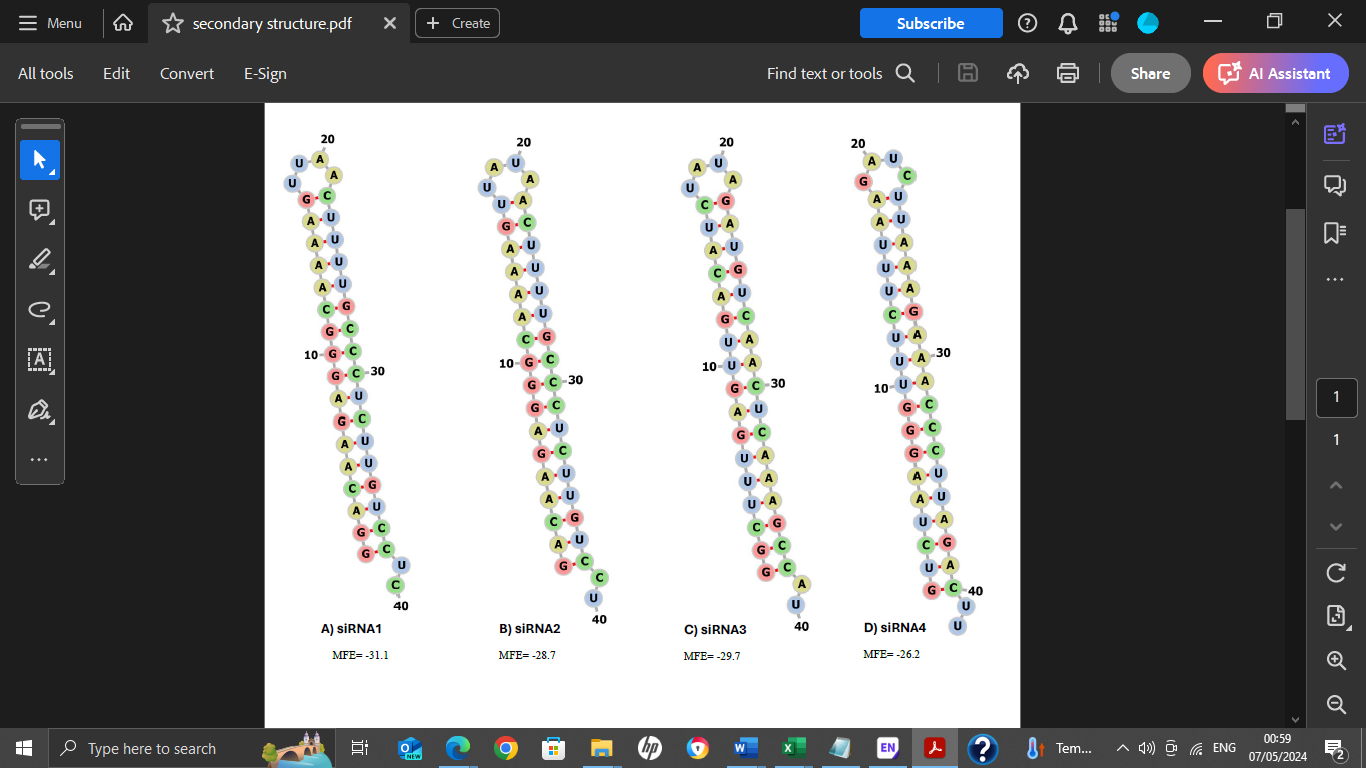


**Additional file 2: Fig. S3: The secondary structure of four siRNAs**

This graphic displays the four siRNAs' minimal free energy (MFE) structures as seen by the RNAfold web server in FORNA viewer. The minimum free energy (MFE) siRNA1(A), siRNA2(B), siRNA3(C), and siRNA4(D) equal -31.1 kcal/mol, -28.7 kcal/mol, -29.7 kcal/mol, and -26.2 kcal/mol respectively.


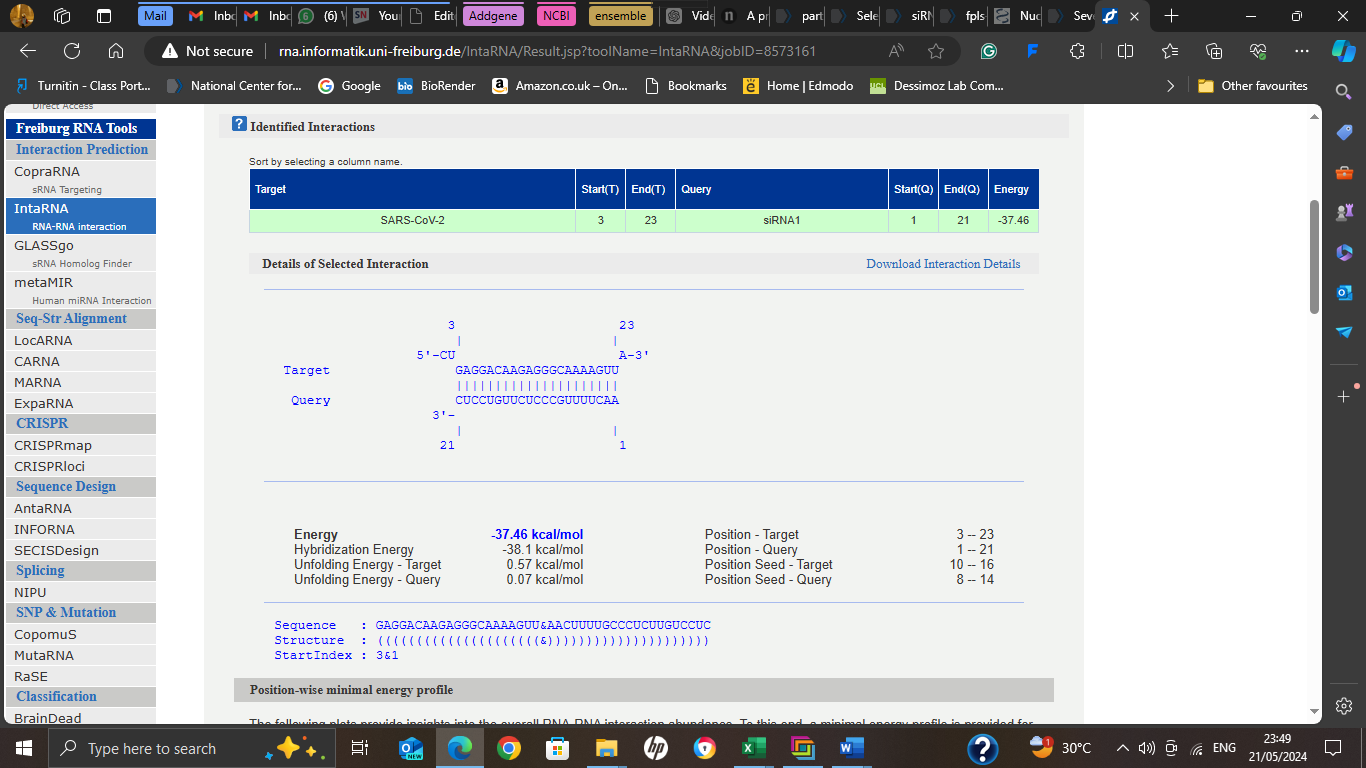


A


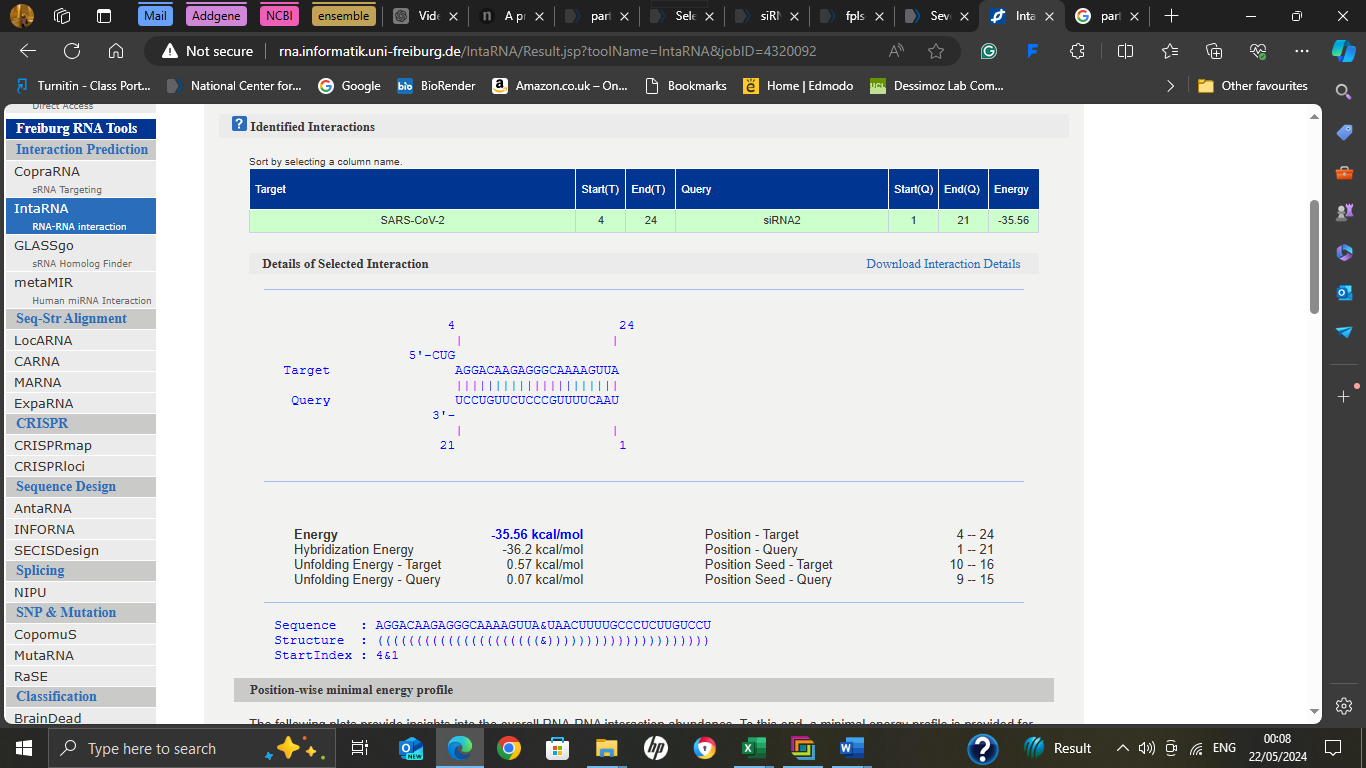


B


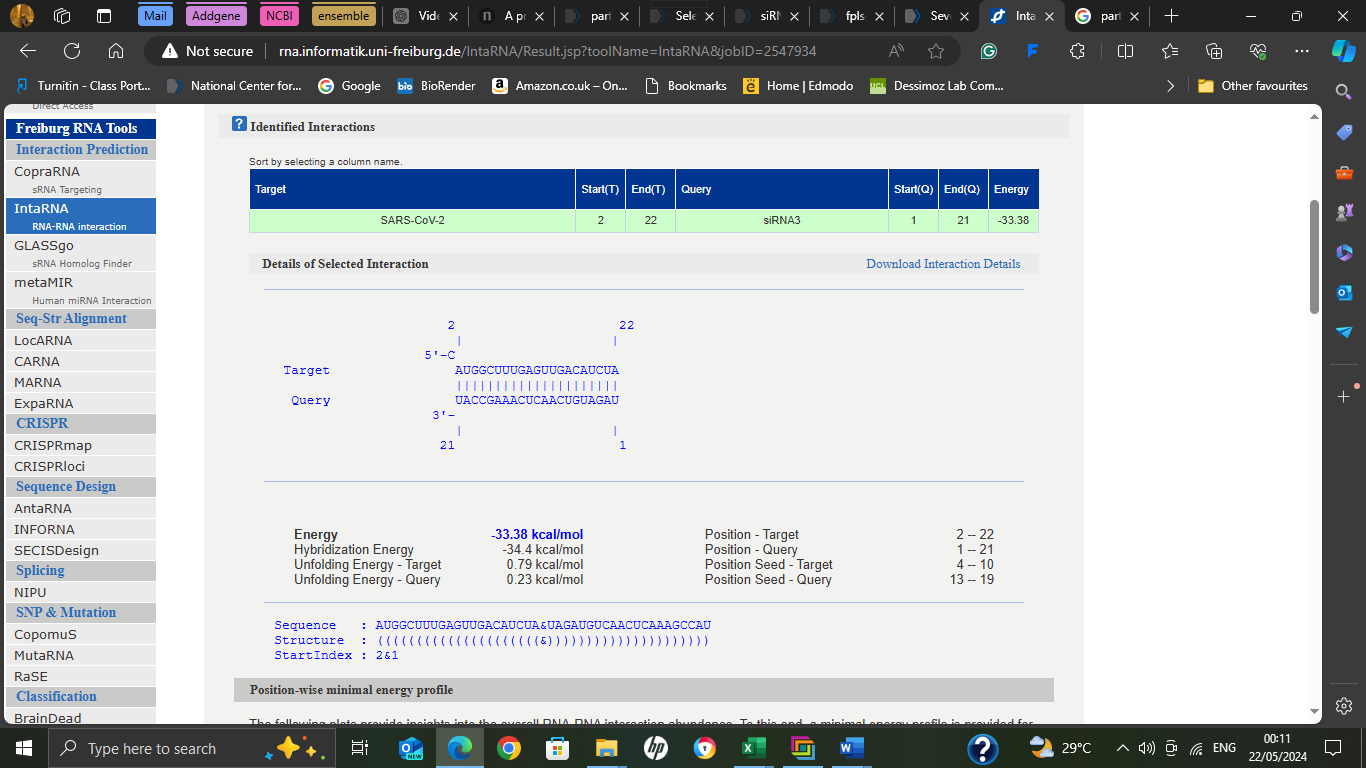


C


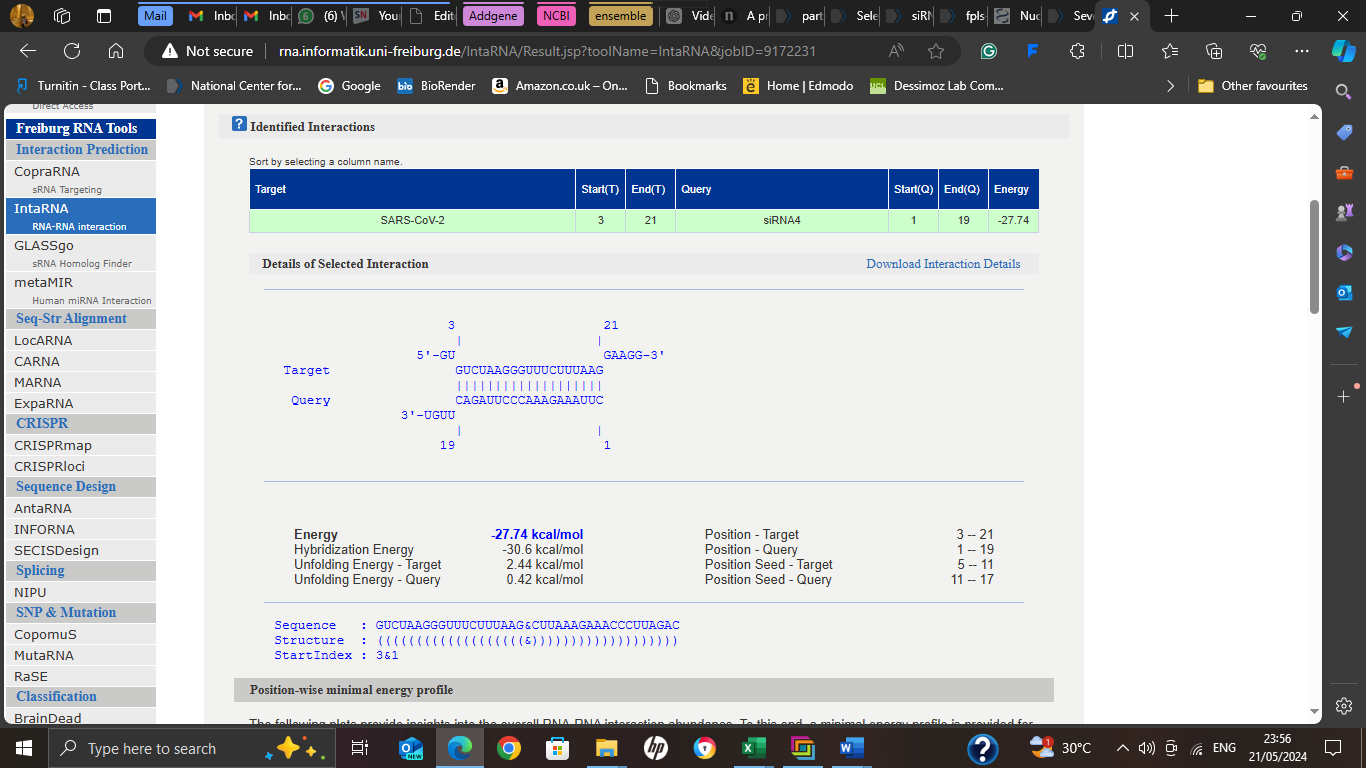


D

**Additional file 2: Fig. S4: The target accessibility of four siRNAs with their targets in SARS-CoV-2 mRNA.**

The four siRNAs' RNA-RNA interactions with the SARS-CoV-2 mRNA are depicted in this figure by the IntaRNA website. A: The kcal/mol of siRNA1 was -37.46. B: The kcal/mol of siRNA2 was -35.56. C:siRNA3 has a kcal/mol of -33.38. C: The -27.74 kcal/mol siRNA4


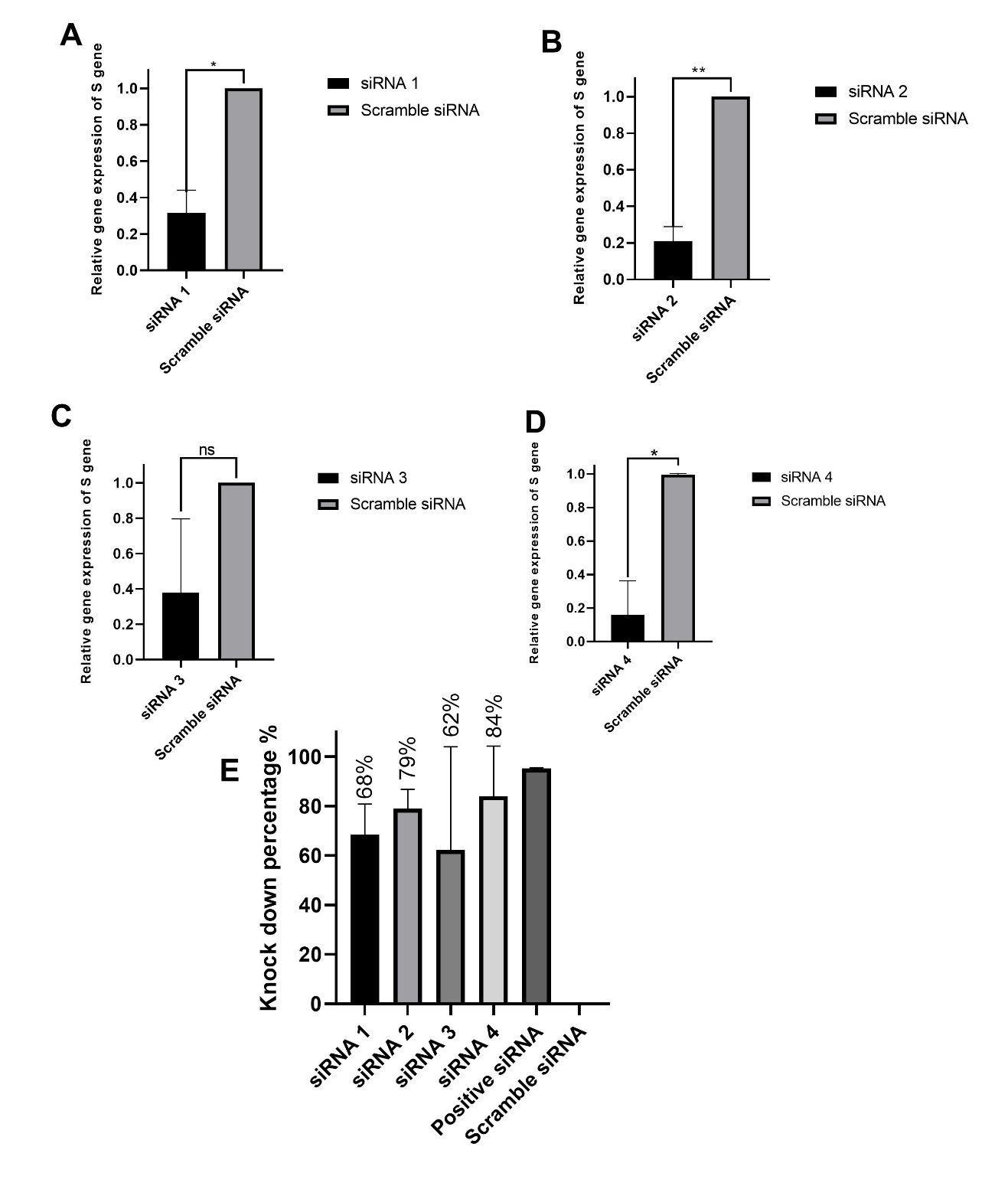


**Additional file 2: Fig. S5: S gene knockdown in VeroE6 cells treated with siRNAs at 12 hours post-infection.**

A: siRNA1 reduced mRNA of the S gene with a P value of ≤0.05 B: siRNA2 demonstrates a significant reduction in mRNA of the S gene with a P value of ≤0.01 C: siRNA3 decreased expression of the S gene without any significance. D: siRNA4 reduced expression of the S gene with a P value of ≤0.05 E: the knockdown percentage of all siRNAs is compared with that of scramble siRNA. All results were normalized against scramble siRNA and quantitatively examined (n = 2 in each group). GraphPad Prism, version 8 was used to represent the values of means ± SEM. Significance was determined using an unpaired t-test. Significant differences are denoted by the symbols

* P≤0.05, ** P≤0.01, *** P≤0.001, **** P≤0.0001, and ns (not significant) ) P>0.05


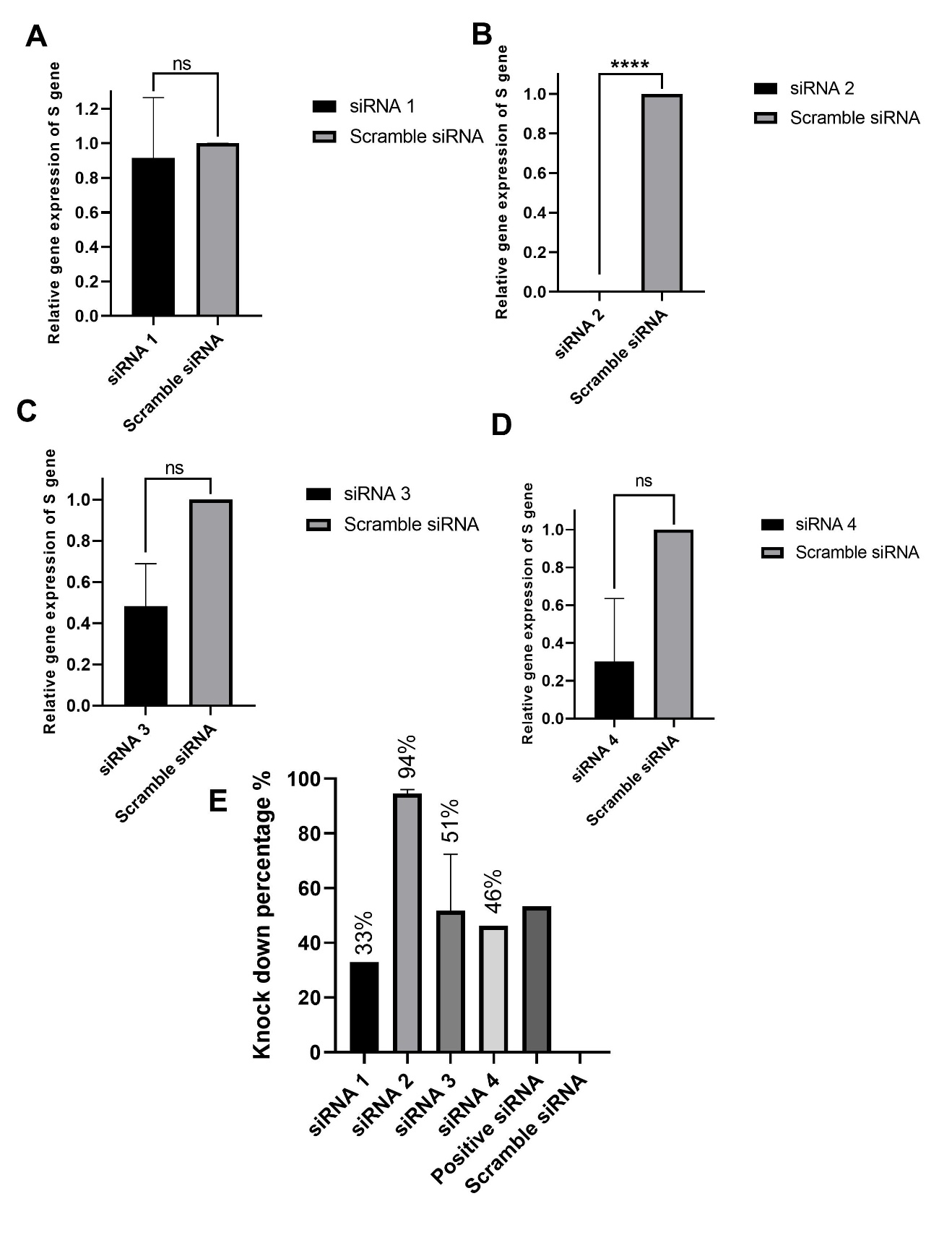


**Additional file 2: Fig. S6: S gene knockdown in VeroE6 cells treated with siRNAs after 36 hours post-infection.**

B: siRNA2 shows high significance in the reduction of mRNA of SARS-CoV-2 with a P value of ≤0.0001 compared to A: siRNA1, C: siRNA3, and D: siRNA4. E: the knockdown percentage of all siRNAs was compared with scramble siRNA. All results were normalized against scramble siRNA and quantitatively examined (n = 2 in each group). GraphPad Prism, version 8 was used to represent the values of means ± SEM. Significance was determined using an unpaired t-test. Significant differences are denoted by the symbols

* P≤0.05, ** P≤0.01, *** P≤0.001, **** P≤0.0001, and ns (not significant) ) P>0.05


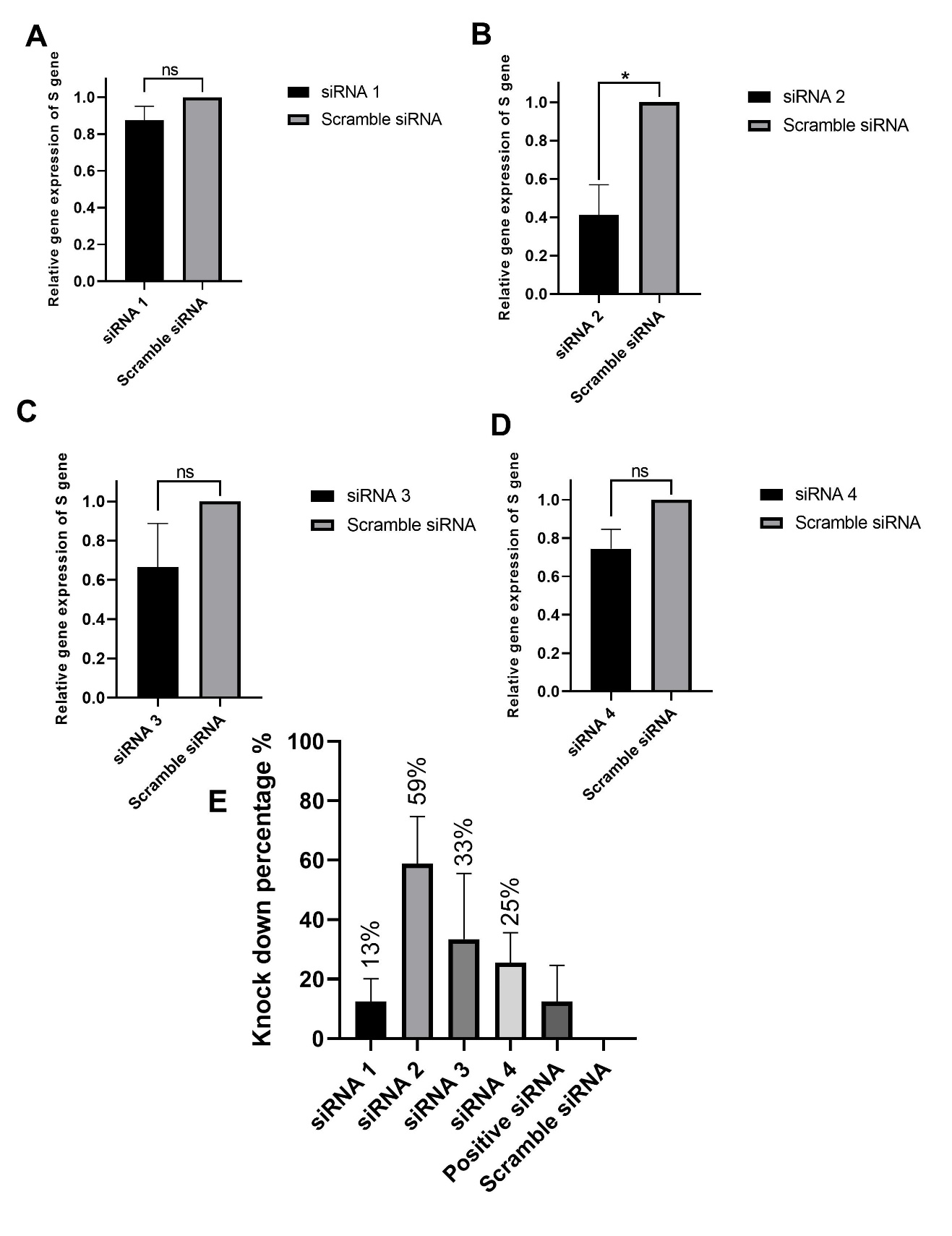


**Additional file 2: Fig. S7: S gene knockdown in VeroE6 cells treated with siRNAs after 48 hours post-infection.**

B: siRNA2 still shows significance in reducing mRNA of the S gene with a P value of ≤ 0.05 compared to A: siRNA1, C: siRNA3, and D: siRNA4. E: the knockdown percentage of all siRNAs was compared with scramble siRNA. All results were normalized against scramble siRNA and quantitatively examined (n = 2 in each group). GraphPad Prism, version 8 was used to represent the values of means ± SEM. Significance was determined using an unpaired t-test. Significant differences are denoted by the symbols

* P≤0.05, ** P≤0.01, *** P≤0.001, **** P≤0.0001, and ns (not significant) ) P>0.05


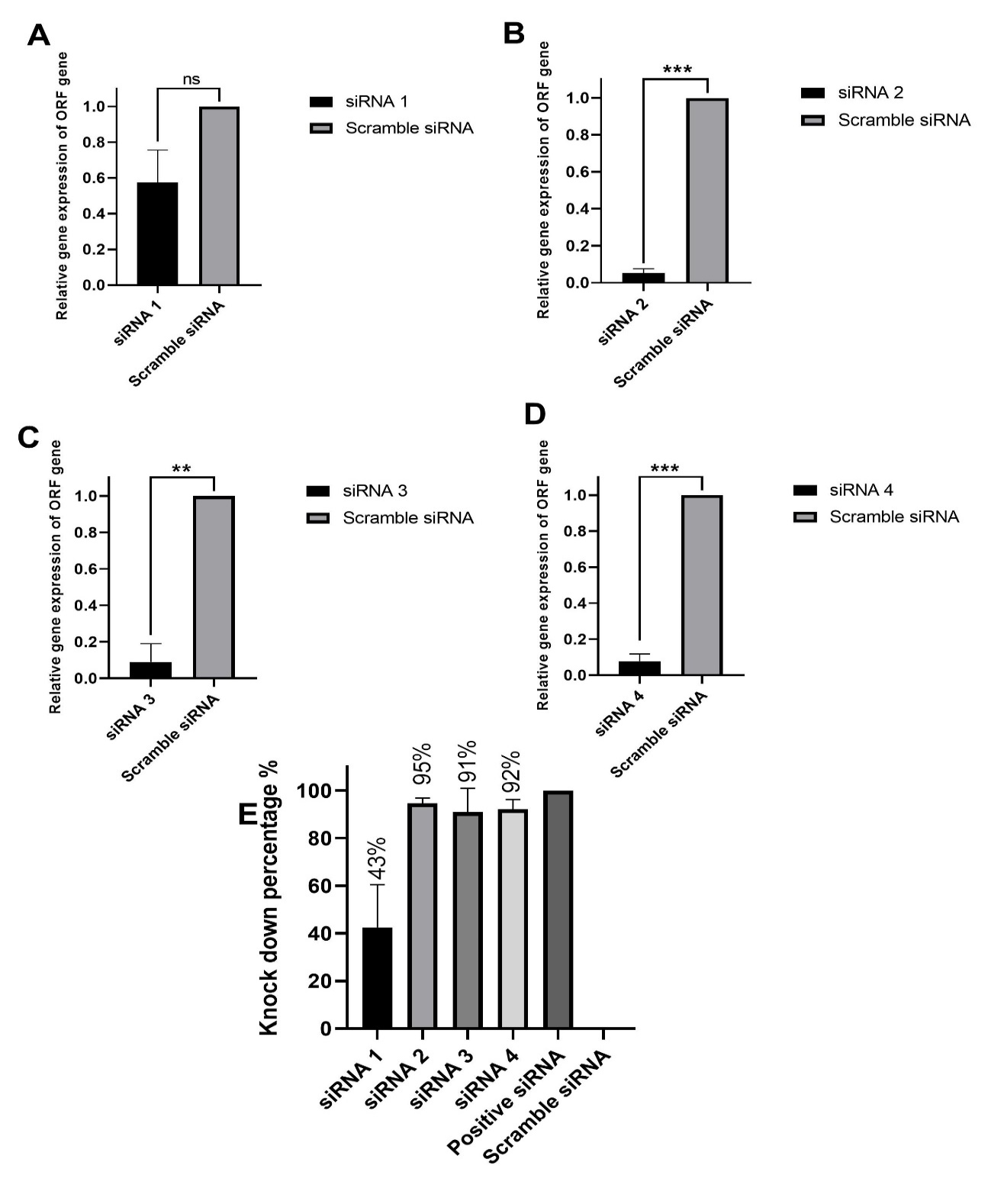


**Additional file 2: Fig. S8: ORF1b gene knockdown in VeroE6 cells treated with siRNAs after 12 hours post-infection.**

A: siRNA1 showed a reduction in mRNA of SARS-CoV-2 with a P value of >0.05, while B: siRNA2, and D: siRNA4 demonstrate a clear decrease in the mRNA of ORF1b with a P value of ≤0.001.Additionally, C:siRNA3 showed a reduction in viral mRNA with a P value of ≤0.01E: the knockdown percentage of all siRNAs is compared with scramble siRNA. All results were normalized against scramble siRNA and quantitatively examined (n = 2 in each group).GraphPad Prism, version 8 was used to represent the values of means ± SEM. Significance was determined using an unpaired t-test. Significant differences are denoted by the symbols

* P≤0.05, ** P≤0.01, *** P≤0.001, **** P≤0.0001, and ns (not significant) ) P>0.05


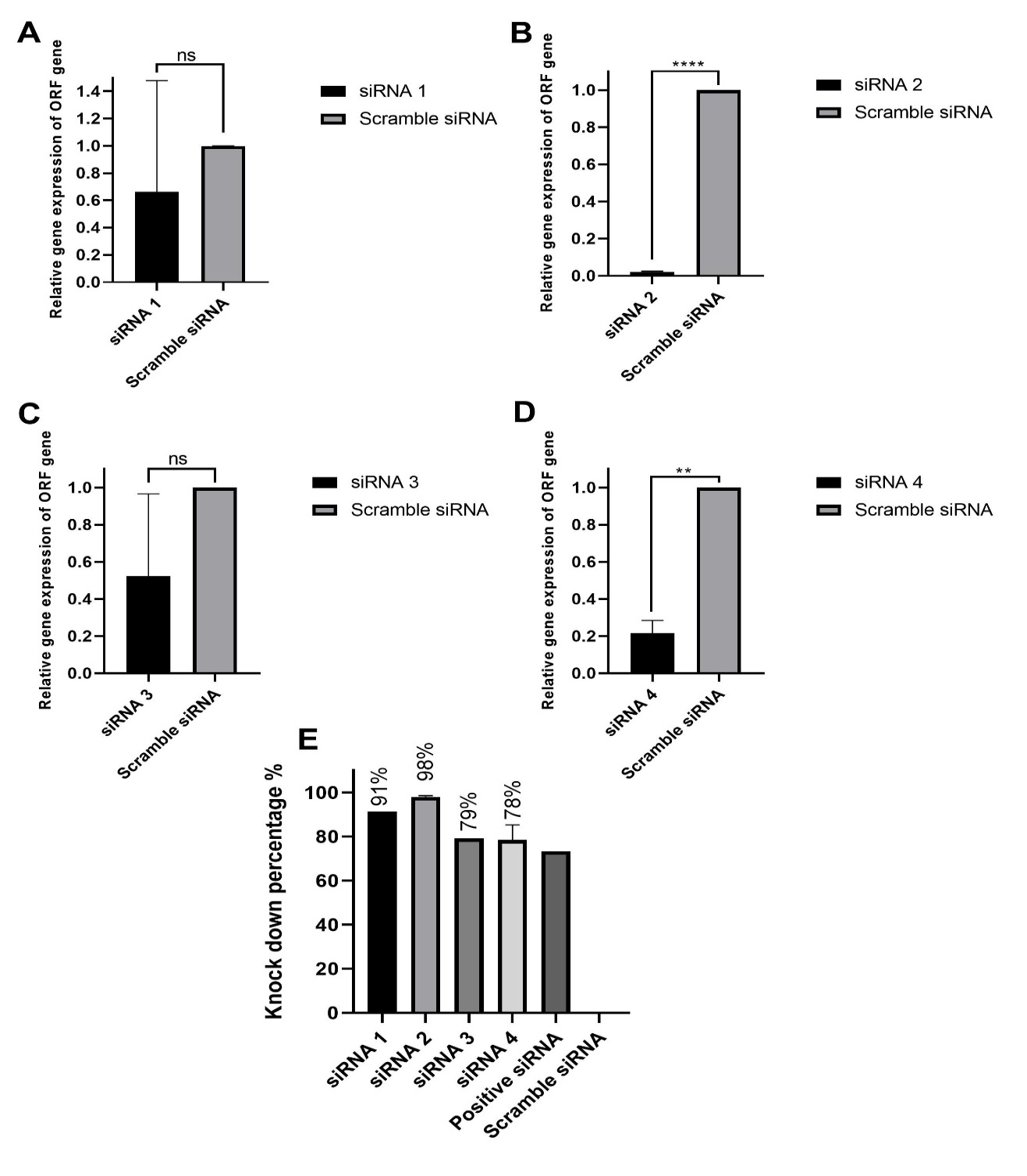


**Additional file 2: Fig. S9: ORF1b gene knockdown in VeroE6 cells treated with siRNAs after 36 hours post-infection.**

A: siRNA1 showed a reduction in ORF1b mRNA with no statistical significance(P>0.05). B: siRNA2 reduced the expression of ORF1b with a P value of ≤0.0001. C:siRNA3 reduced the expression of ORF1b with no statistical significance. D: siRNA4 shows a decrease in the mRNA of ORF1b with a P value of ≤0.01. E: the knockdown percentage of all siRNAs compared with scramble siRNA. All results were normalized against scramble siRNA and quantitatively examined (n = 2 in each group).

GraphPad Prism, version 8 was used to represent the values of means ± SEM. Significance was determined using an unpaired t-test. Significant differences are denoted by the symbols

* P≤0.05, ** P≤0.01, *** P≤0.001, **** P≤0.0001, and ns (not significant) ) P>0.05


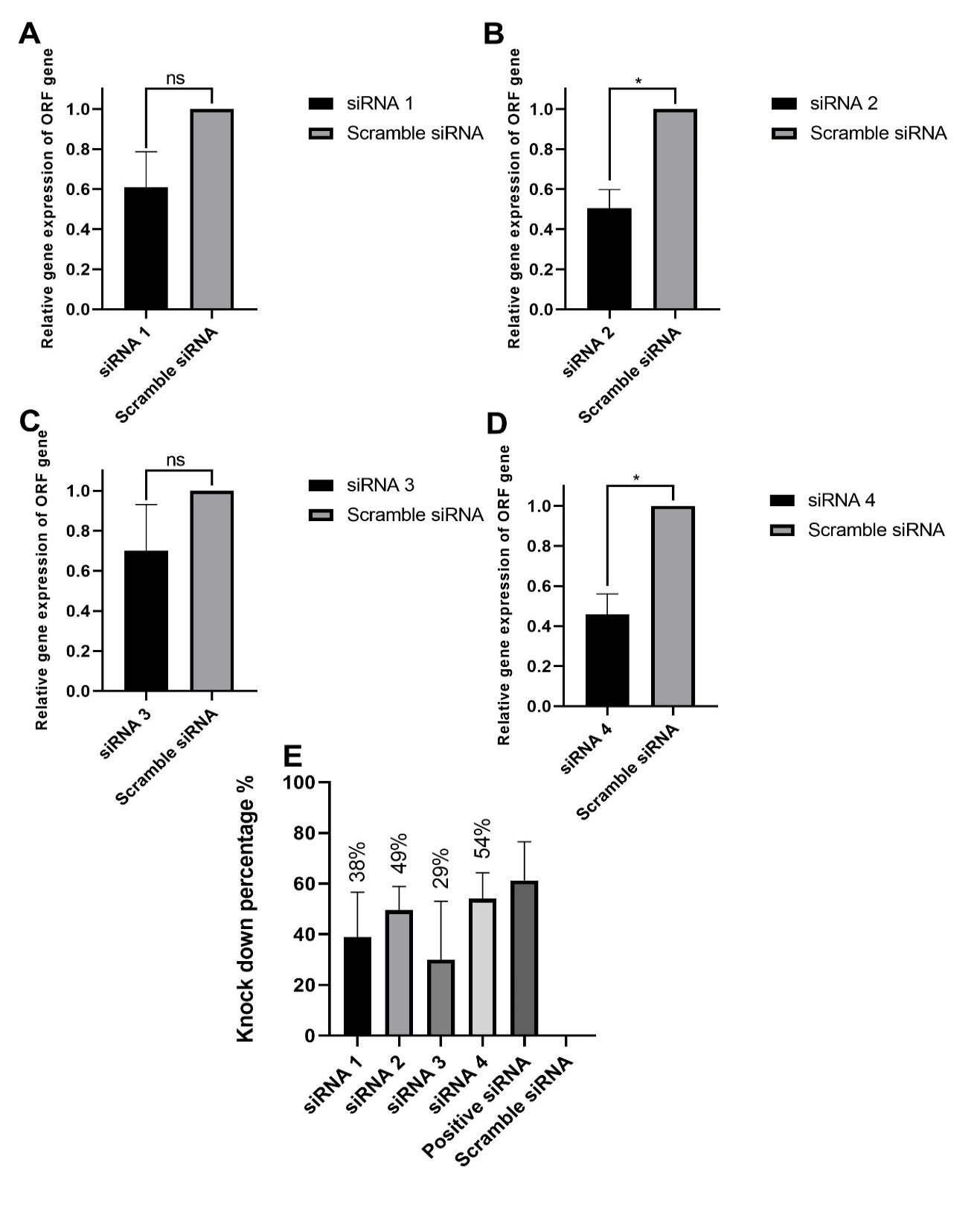


**Additional file 2: Fig. S10: ORF1b gene knockdown in VeroE6 cells treated with siRNAs after 48 hours post-infection.**

B: siRNA2, and D: siRNA4 both demonstrate a clear decrease in ORF1b mRNA with a P value of P≤0.05. In contrast A: siRNA1 and C:siRNA3 show a reduction in ORF1b mRNA without statistical significance. E: the knockdown percentage of all siRNAs compared to scramble siRNA. All results were normalized against scramble siRNA and quantitatively examined (n = 2 in each group). GraphPad Prism, version 8 was used to represent the values of means ± SEM. Significance was determined using an unpaired t-test. Significant differences are denoted by the symbols

* P≤0.05, ** P≤0.01, *** P≤0.001, **** P≤0.0001, and ns (not significant) ) P>0.05

**Additional file 2:** Table S1 : Primers used in RT-PCR

| Name | Sequence |
| --- | --- |
| F-S_COV2-NRC | TACCCATTGGTGCAGGTATATGC |
| R-S_COV2-NRC | GTGTAGGCAATGATGGATTGACTA |
| ORF1F*^a^ | TGGGGYTTTACRGGTAACT |
| ORF1R*^a^ | AACRCGCTTAACAAAGCA |
| GAPDH-F*b | CCTCCACCTTTGACGCT |
| GAPDH-R*b | TTGCTGTAGCCAAATTCGTT |

a: Chu, D.K.W., et al., *Molecular Diagnosis of a Novel Coronavirus (2019-nCoV) Causing an Outbreak of Pneumonia.* Clin Chem, 2020. **66**(4): p. 549-555.

b: List, W., et al., *Occurrence of SARS-CoV-2 in the intraocular milieu.* Experimental Eye Research, 2020. **201**: p. 108273.
